# Differential location of NKT and MAIT cells within lymphoid tissue

**DOI:** 10.1038/s41598-022-07704-4

**Published:** 2022-03-08

**Authors:** Darryl N. Johnson, Zheng Ruan, Emma V. Petley, Sapna Devi, Lauren E. Holz, Adam P. Uldrich, Jeffrey Y. W. Mak, Jyh Liang Hor, Scott N. Mueller, James McCluskey, David P. Fairlie, Phillip K. Darcy, Paul A. Beavis, William R. Heath, Dale I. Godfrey

**Affiliations:** 1 Department of Microbiology and Immunology at the Peter Doherty Institute for Infection and Immunity, The University of Melbourne, Parkville, VIC 3010 Australia; 2 Australian Research Council Centre of Excellence for Advanced Molecular Imaging, University of Melbourne, Parkville, VIC 3010 Australia; 3 Cancer Immunology Program, Peter MacCallum Cancer Centre, Melbourne, VIC 3000 Australia; 4 Australian Research Council Centre of Excellence in Advanced Molecular Imaging, Institute for Molecular Bioscience, The University of Queensland, Brisbane, QLD 4072 Australia; 5 Sir Peter MacCallum Department of Oncology, The University of Melbourne, Parkville, VIC 3010 Australia

**Keywords:** Imaging the immune system, T cells, NKT cells

## Abstract

Natural Killer T (NKT) cells and Mucosal-Associated Invariant T (MAIT) cells are innate-like T cells that express semi-invariant αβ T cell receptors (TCRs) through which they recognise CD1d and MR1 molecules, respectively, in complex with specific ligands. These cells play important roles in health and disease in many organs, but their precise intra-organ location is not well established. Here, using CD1d and MR1 tetramer staining techniques, we describe the precise location of NKT and MAIT cells in lymphoid and peripheral organs. Within the thymus, NKT cells were concentrated in the medullary side of the corticomedullary junction. In spleen and lymph nodes, NKT cells were mainly localised within T cell zones, although following in vivo activation with the potent NKT-cell ligand α-GalCer, they expanded throughout the spleen. MAIT cells were clearly detectable in Vα19 TCR transgenic mice and were rare but detectable in lymphoid tissue of non-transgenic mice. In contrast to NKT cells, MAIT cells were more closely associated with the B cell zone and red pulp of the spleen. Accordingly, we have provided an extensive analysis of the in situ localisation of NKT and MAIT cells and suggest differences between the intra-organ location of these two cell types.

## Introduction

Natural Killer T (NKT) and Mucosal-Associated Invariant T (MAIT) cells are two distinct classes of unconventional αβ T cells that recognise non-peptide antigens in complex with MHC class-I-like molecules. Type I (sometimes known as invariant) NKT cells express a semi-invariant TCRα chain (Vα 14Jα18 in mice; Vα24Jα18 in humans) that pairs with a biased pool of TCRβ chains through which they recognise self and foreign glycolipids presented in complex with CD1d^1^. The prototypical antigen recognised by type I NKT cells is the microbial antigen α-galactosylceramide (α-GalCer)^2^. From here, the term NKT cells is referring to type I NKT cells.

Similar to NKT cells, MAIT cells also express an invariant TCRα chain (Vα19Jα33 in mice; Vα7.2Jα33 in humans) paired with an oligoclonal pool of TCRβ chains^3–5^. Unlike NKT cells however, MAIT cells recognise microbial-derived vitamin B2-based metabolites, such as 5-OP-RU (5-(2-oxopropylideneamino)-6-d-ribitylaminouracil), presented in complex with MR1 molecules^3–5^. Following activation, NKT cells and MAIT cells rapidly produce a broad range of cytokines and play protective roles against microbial infections and cancers as well as pathogenic effects in autoimmune and allergic diseases^1,5^. However, our knowledge of the in situ locations of NKT cells and MAIT cells is very limited.

NKT and MAIT cells develop in the thymus and migrate to the periphery, where they can be found in a broad range of both secondary lymphoid and peripheral tissues and organs including liver, intestines, lungs, skin, adipose tissue and other sites^5,6^. Early studies to identify NKT cells in situ used a CXCR6-GFP reporter system because NKT cells were highly enriched within the CXCR6-GFP^+^ fraction of T cells. This reporter was used to visualise NKT cells patrolling liver sinusoids and interacting with Kupffer cells^7,8^, responding to *Borrelia burgdorferi* in knee joints^9^ or residing in the medulla of LNs^10^. A limitation with this system is that not all CXCR6-GFP^+^ cells are NKT cells and not all NKT cells express CXCR6-GFP^8^. Adoptive transfer of CFSE-labelled Vα14 transgenic NKT cells into non-transgenic C57BL/6 (B6) recipients provided some of the first images of NKT cells which were detected in LN paracortex and splenic T cell zones (TCZ) of the white pulp (WP)^11,12^. However, it is unclear whether adoptively transferred TCR transgenic NKT cells will migrate and localise to the same sites as endogenous non-transgenic NKT cells. Immunohistology staining for a combination of CD3 or TCRβ and NK1.1 allowed for the first description of endogenous NK1.1^+^ T cells and located these cells predominantly to the red pulp (RP) and marginal zone of the spleen^13^ and liver sinusoids^14^. A limitation with this approach is the fact that not all NK1.1^+^ T cells are NKT cells and not all NKT cells are NK1.1^+^^15^. CD1d-α-GalCer tetramer staining is the best way to accurately identify NKT cells and has been used with immunohistology to detect endogenous NKT cells in Vα14 TCR transgenic mice where NKT cells are highly abundant^16^. More recently, this approach has been used to detect endogenous NKT cells in thymus, spleen, lymph nodes and adipose tissue^17–20^. Within thymus, most NKT cells were detected throughout the medulla. In peripheral lymphoid organs, the location of NKT cells may be strain dependent. Thus, in the spleen of B6 mice, most NKT cells were found in the RP, while conversely, in the spleen of BALB/c mice, the major location of NKT cells shifted towards the TCZ^17,18^. Similarly, CD1d tetramer staining has located NKT cells within the LN follicle^18^ or intrafollicular space^19^ in B6 mice, but LN paracortex in BALB/c mice^18^. Thus, discrepancies between reports on of NKT cell locations in peripheral organs may reflect differences in the techniques for detection as well as strain-dependent differences in location of NKT cell subsets.

In the case of MAIT cells, immunohistological staining for CD3, CD8, Vα7.2, CD161 and/or IL-18R has been used to study MAIT cells in human liver^21–25^, intestine^22,26–29^, pancreas^30^, brain^31–33^ and lymph nodes^22^ in either heathy or diseased states. However, these surrogate markers are not limited to MAIT cells^5^, so it is difficult to know if the cells detected were MAIT or MAIT-like cells. Recently, direct MAIT cell staining with MR1-5-OP-RU tetramer, the gold standard for MAIT cell detection, was used to detect MAIT cells in mouse lung, a site with a high proportion of MAIT cells, following *Legionella longbeachae* infection^34^. The location of MAIT cells, defined by MR1-5-OP-RU tetramer, in other tissues remains unknown.

Because there remains great uncertainty about the in situ localisation of NKT and MAIT cells, in this study, we have investigated this problem using CD1d-α-GalCer and MR1-5-OP-RU tetramers, which are the gold standard for identifying NKT and MAIT cells, in a range of different mouse tissues, including appropriate control tetramers and CD1 and MR1 deficient mice, to increase confidence that the cells detected are indeed NKT and MAIT cells. We have clarified and expanded upon previous studies in terms of NKT cell location, including following in vivo activation and compared the locational differences between NKT cells and MAIT cells in secondary lymphoid organs.

## Results

### Identification of endogenous NKT cells in situ with α-GalCer-loaded CD1d tetramers

**Figure 1:**
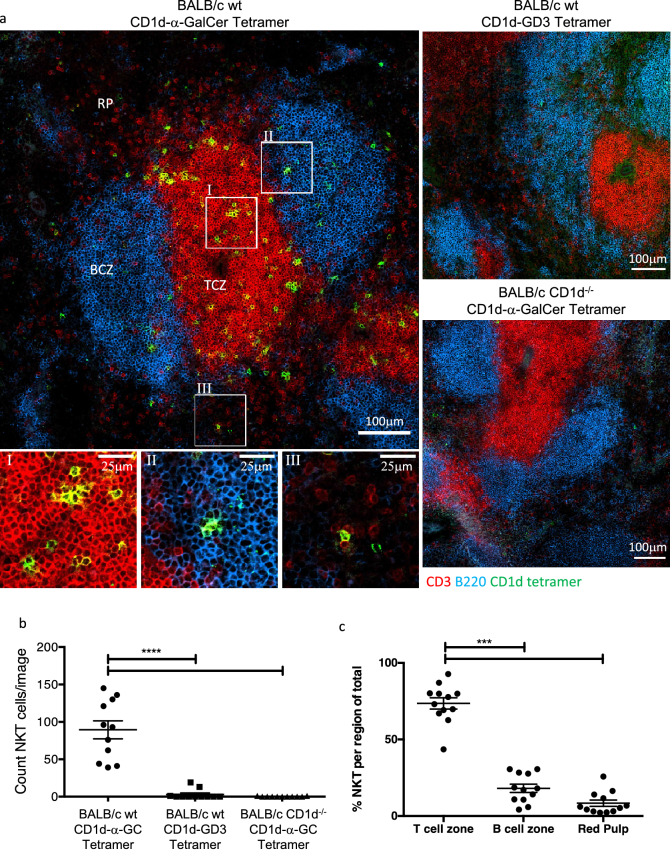

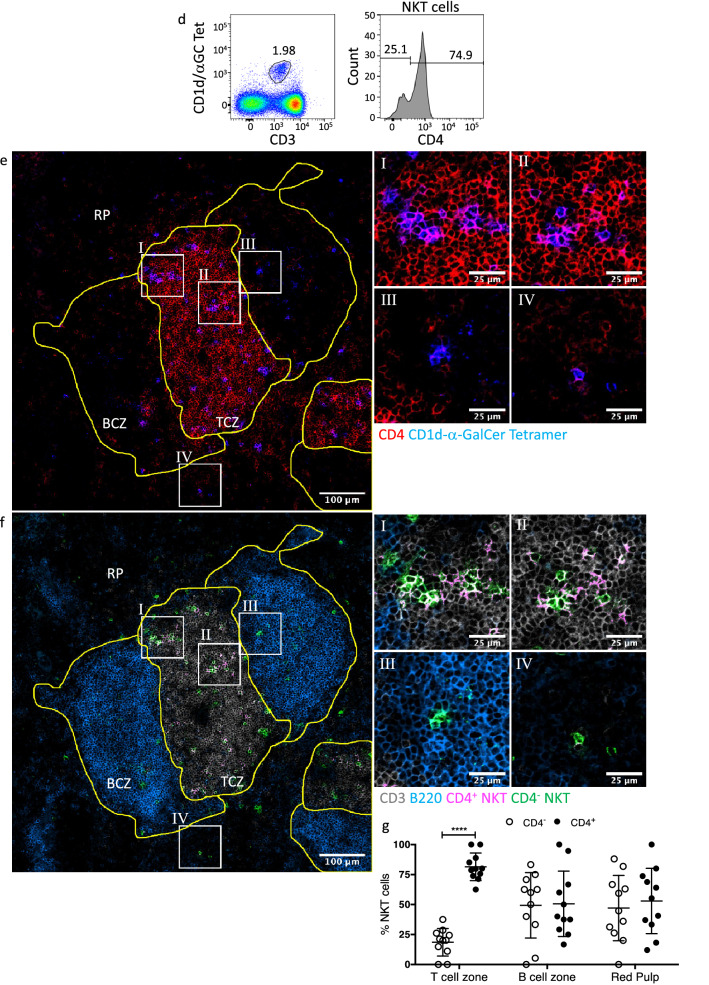
Identification of NKT cells in situ with CD1d-α-GalCer tetramer within BALB/c spleen. Spleen sections from BALB/c WT or CD1d^−/−^ mice stained with CD1d tetramers loaded with α-GalCer or GD3 and anti-B220, CD3 and CD4 antibodies. (**a**) Representative images showing CD1d tetramer (green), B220 (blue) and CD3 (red) staining within splenic red pulp (RP), B cell zone (BCZ) and T cell zone (TCZ). (**b**) Count of CD1d-α-GalCer tetramer^+^CD3^+^ NKT cells per 0.63 mm^2^ image and (**c**) proportion of NKT cells within each region of the total number of NKT cells per image, one-way ANOVA with Tukey post-hoc test. (**d**) Representative FACS plot and histogram showing percentage of NKT cells of total T cells and CD4 expression on NKT cells. Cells gated on live B220^−^ lymphocytes, Tet = Tetramer. (**e**) Representative image with corresponding zoomed regions of showing CD4 (red) and CD1d-α-GalCer tetramer (blue). (**f**) Representative image of those CD4^+^ (purple) or CD4^−^ (green) NKT cells as determined by colocation analysis, CD3 (gray) and B220 (blue). (**g**) Mean proportion of CD4^+^ to CD4^−^ NKT cells within each splenic region. Image scale bars 100 μm with corresponding zoomed regions of interest scale bar 25 μm. Microscopy images acquired with Zen 2012 (ZEISS ZEN Microscope Software for Microscope Components) and processed with FIJI/ImageJ (Fiji (imagej.net)) and Imaris 9.3 (Microscopy Image Analysis Software—Imaris—Oxford Instruments (oxinst.com)) and figure compiled with Microsoft PowerPoint (Microsoft PowerPoint Slide Presentation Software|Microsoft 365). Data n = 12 mice combined from 3 independent experiments, mean ± SEM, two-way ANOVA with Sidak post-hoc test, *** = *p* < 0.005, **** = *p* < 0.001.

We have used CD1d tetramer for immunohistological analysis of NKT cells in a range of mouse tissues. To validate the specificity of our approach, fresh frozen spleen sections from BALB/c wild type (WT) or BALB/c.CD1d^−/−^ mice were stained with CD1d tetramers loaded with α-GalCer or left unloaded (presumably carrying endogenous lipids) (Fig. 1a and Supplementary Fig. S1). Within the WT spleen, cells could be identified that had clearly bound α-GalCer-loaded CD1d tetramer with limited staining observed on WT sections stained with unloaded CD1d tetramer or CD1d^−/−^ mouse spleen sections stained with CD1d-α-GalCer tetramer. Though, in all three groups, we observed rare examples of CD1d tetramer staining that appeared not to associate with the TCRβ stain (Supplementary Fig. S1b), thus co-localisation of CD1d tetramer and TCRβ was investigated. Voxel plots of the three groups indicated a clear association within BALB/c WT sections of voxels stained with CD1d-α-GalCer and TCRβ (Supplementary Fig. S1c). Analysis of Pearson’s correlation and the area co-stained by CD1d-α-GalCer tetramer and TCRβ both indicated a positive correlation between CD1d-α-GalCer and TCRβ staining on WT sections compared to negative control-stained sections (Supplementary Fig. S1d and e). Taken together, these data highlight the ability of the CD1d-α-GalCer tetramers to specifically stain endogenous NKT cells in situ. Importantly, they also demonstrate the need to co-stain with T cell-specific markers such as TCRβ or CD3 and include negative controls for stain and tissue to best eliminate, albeit rare, non-NKT cell staining from analysis.

Having established the technique to reliably identify NKT cells in situ, we next examined the location of NKT cells in primary and secondary lymphoid organs. On average, roughly 100 NKT cells per field of view could be observed within BALB/c spleen (Fig. 1b), the majority (~ 70%) of which were found within the TCZ rather than the BCZ or RP (Fig. 1c). Within these regions, NKT cells were scattered either alone or in small clusters. We also examined spleens from C57BL/6 (B6) mice and detected roughly 40 NKT cells per field of view (Supplementary Fig. S2a and b). There was also a clear difference in the location of NKT cells between the two strains of mice. Where in the spleens of B6 mice, more NKT cells were found in the RP with the remaining NKT cells in roughly equal proportions in the TCZ and BCZ (Supplementary Fig. S2c). In spleens from both BALB/c and B6 mice, we detected some CD1d tetramer^+^ cells that are not likely to be NKT cells because they did not co-stain for CD3/TCR. Similar infrequent staining was observed in CD1d^−/−^ spleens and WT spleens stained with CD1d tetramer loaded with a negative control glycolipid antigen, disialoganglioside (GD3) (Fig. 1a, Supplementary Fig. S2d).

The distribution of NKT cell subsets in BALB/c mice was also investigated based on the expression or absence of CD4, which defines many, but not all NKT cells, as shown by flow cytometry (Fig. 1d). In general, CD4 expression in the spleen was observed throughout the organ, though as expected the greatest concentration of expression was associated with CD3 within the TCZ (Fig. 1e), representing conventional CD4 T cells. Using colocation analysis between CD3^+^CD1d-α-GalCer^+^ and CD4^+^ stains to separate NKT cells into CD4 expressor and non-expressor channels, the distribution of CD4^+^ and CD4^−^ NKT cells could be determined (Fig. 1f). Interestingly, while the majority (~ 75%) of NKT cells within the TCZ expressed CD4, there were roughly equal proportions of CD4^+^ and CD4^−^ NKT cells within the RP or BCZ expressed this marker (Fig. 1g). These data suggest that there is differential distribution of NKT CD4^+^ and CD4^−^ subsets may reflect differential expression of chemokine receptors or adhesion molecules^35,36^.

**Figure 2:**
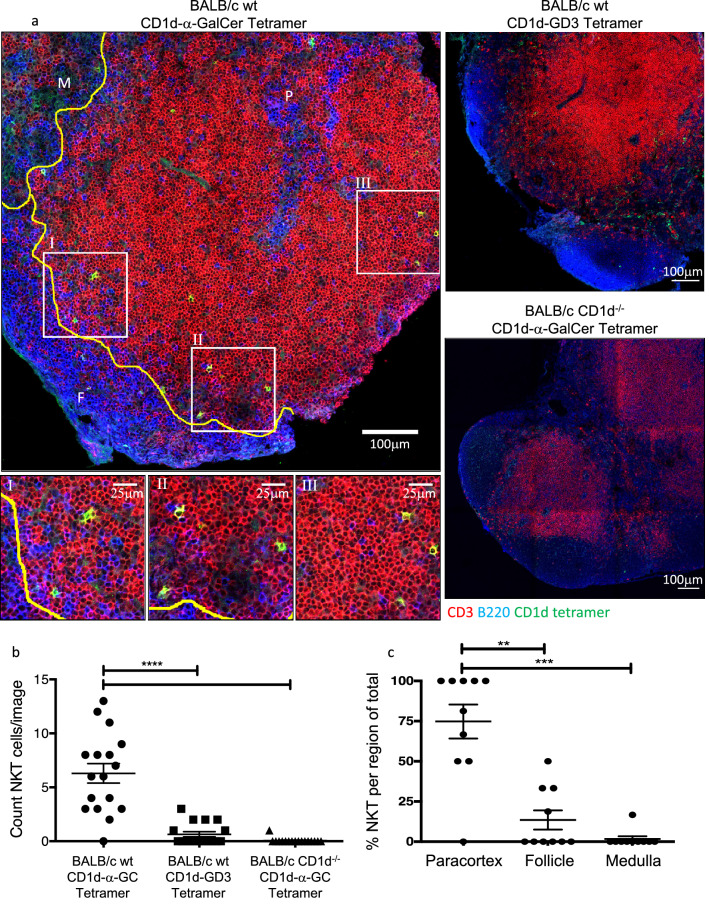
Distribution of NKT cells within the mouse Lymph Node. Sections of brachial lymph nodes from BALB/c WT mice stained with α-GalCer- or GD3-loaded CD1d tetramers and anti-B220 and CD3 antibodies. (**a**) Representative image showing CD1d-α-GalCer tetramer (green), B220 (blue) and CD3 (red) staining within lymph node follicle (F), paracortex (P) and medulla (M), scale bar 100 μm, with corresponding zoomed regions of interest, scale bar 25 μm. (**b**) Total count of NKT cells per 0.63 mm^2^ image and, (**c**) proportion of cells within each lymph node region. Microscopy images acquired with Zeiss Zen 2012 (ZEISS ZEN Microscope Software for Microscope Components) and processed with FIJI/ImageJ (Fiji (imagej.net)) and Imaris 9.3 (Microscopy Image Analysis Software—Imaris—Oxford Instruments (oxinst.com)) and figure compiled with Microsoft PowerPoint (Microsoft PowerPoint Slide Presentation Software | Microsoft 365). Data n = 12 mice combined from 3 independent experiments, mean ± SEM, Friedman with Dunn’s post-hoc test, ** = *p* < 0.01, *** = *p* < 0.005, **** = *p* < 0.001.

Within peripheral (brachial) lymph nodes (LN) there were far fewer NKT cells observed per field of view than in spleen sections, nonetheless, compared to negative controls NKT cells could still be observed (Fig. 2a, b). By scanning multiple sections, we were able to determine that the majority of NKT cells were localised within the paracortex rather than the medulla or B cell follicles of lymph nodes (Fig. 2c).

**Figure 3:**
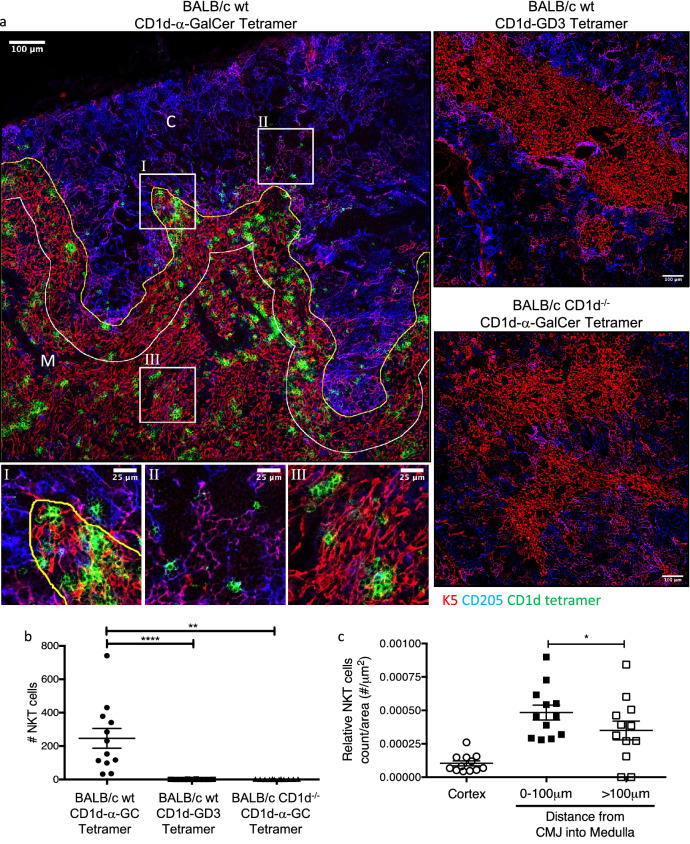

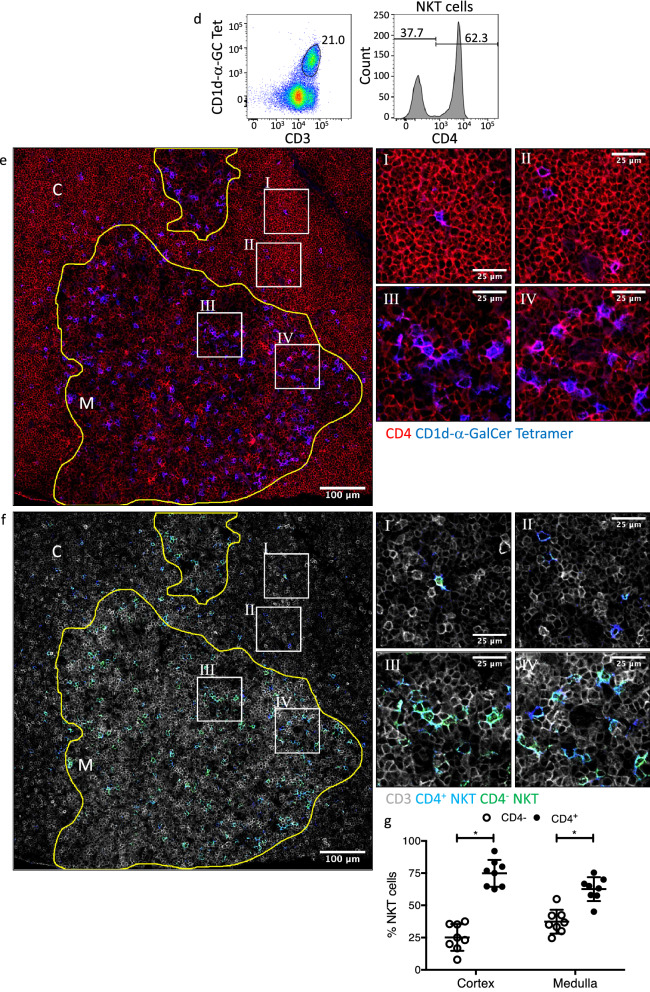
Distribution of NKT cells within the mouse thymus. Thymus sections from BALB/c WT mice stained with α-GalCer or GD3 loaded CD1d tetramers and anti-CD205 and cytokeratin-5 or anti-CD4 and CD3 antibodies. (**a**) Representative image showing CD1d-α-GalCer tetramer (green), CD205 (blue) and cytokeratin-5 (red) staining within thymic cortex (C) and medulla (M). White line within medulla shows distance 100 μm from corticomedullary junction. (**b**) Total count of NKT cells per 0.63 mm^2^ image, Friedman test with Dunn’s comparison, and (**c**) proportion of NKT cells within each thymic region, Wilcoxon test. (**d**) Representative FACS plot and histogram showing percentage of NKT cells of total T cells and CD4 expression on NKT cells. Thymocytes were CD24 depleted and gated on live B220^−^ lymphocytes, Tet = Tetramer. (**e**) Representative image with corresponding zoomed regions of showing CD4 (red) and CD1d-α-GalCer tetramer (green). (**f**) Representative image of CD4^+^ (purple) or CD4^−^ (green) NKT cells as determined by colocation analysis, CD3 (gray). (**g**) Mean proportion of CD4^+^ to CD4^−^ NKT cells within each thymic region, two-way ANOVA with Sidak post-hoc test. Image scale bar 100 μm with corresponding zoomed regions of interest, scale bar 25 μm. Microscopy images acquired with Zen 2012 (ZEISS ZEN Microscope Software for Microscope Components) and processed with FIJI/ImageJ (Fiji (imagej.net)) and Imaris 9.3 (Microscopy Image Analysis Software—Imaris—Oxford Instruments (oxinst.com)) and figure compiled with Microsoft PowerPoint (Microsoft PowerPoint Slide Presentation Software|Microsoft 365). Data n = 8 mice combined from 2 independent experiments, mean ± SEM, * = *p* < 0.05.

The location of NKT cells in thymus was examined by costaining for thymic cortical and medullary regions using CD205, cytokeratin 5 and CD3 and CD1d-α-GalCer (Fig. 3a, b). This demonstrated that, consistent with previous reports^18^ the vast majority of NKT cells were located within the medulla. Furthermore, the medullary NKT cells tended to localise close to, or within, the corticomedullary junction (CMJ) as a higher density of these cells were observed within 100 μm of the CMJ (Fig. 3c). Thymic NKT cells can also be subdivided into CD4^+^ and CD4^−^ fractions, as shown by flow cytometry (Fig. 3d) and immunohistology (Fig. 3e). Following co-localisation analysis (Fig. 3f, g), we found that within both thymic regions, most NKT cells expressed CD4 (~ 75% within the cortex and ~ 60% within the medulla).

These results demonstrate that NKT cells occupy specific locations within the primary and secondary lymphoid organs and, for the most part, they were located in similar regions to conventional T cells in BALB/c mice. We have also shown that while NKT can be divided into CD4^+^ and CD4^−^ subsets, for the most part both populations were intermingled, although there were some differences in the ratio of these subsets in different locations.

### Thymic medullary structure in mice lacking NKT cells

**Figure 4:**
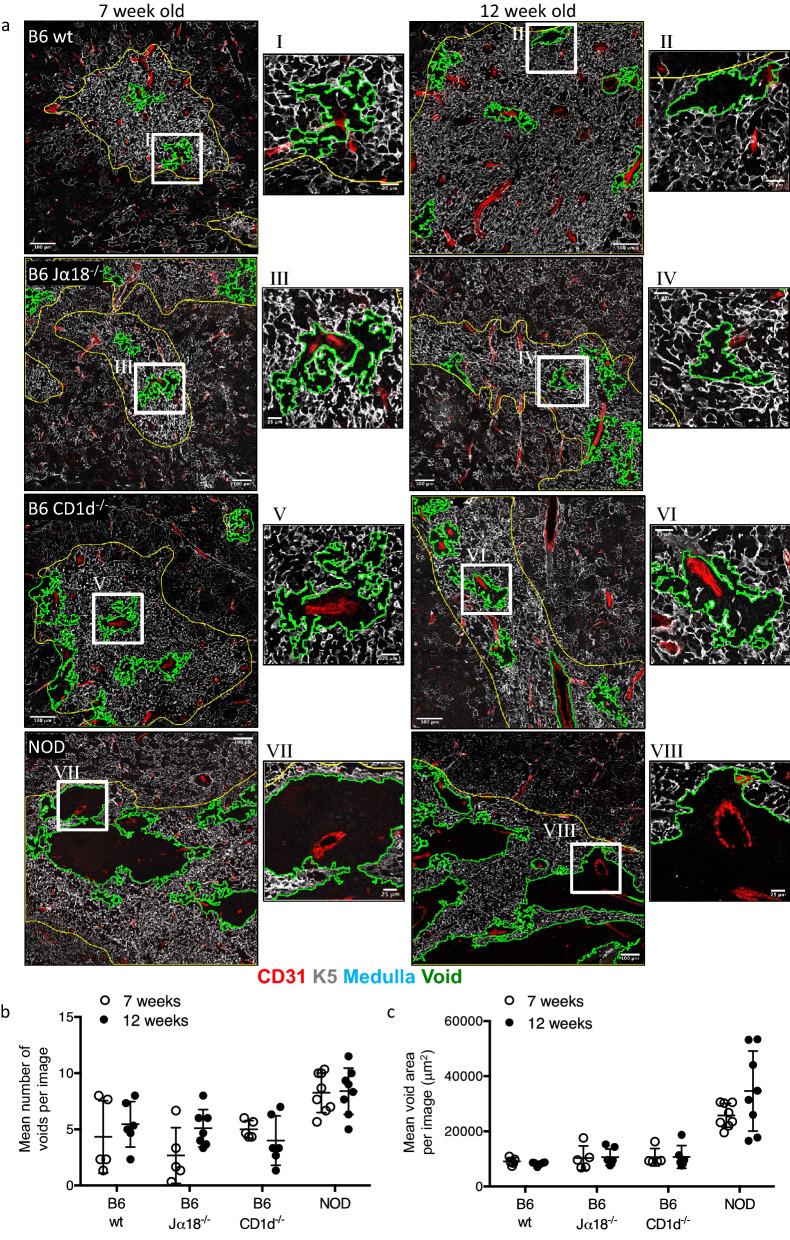
No evidence of an increase in medullary voids in thymuses of NKT cell-deficient mice. B6 WT, B6 Jα18^−/−^, B6 CD1d^−/−^ and NOD thymus sections stained with CD31 (Red), cytokeratin 5 (K5), (Gray) and DAPI (not shown). (**a**) Representative image showing medulla (yellow line) and medullary epithelial cell free areas (voids, green lines). Medulla determined from the K5 and DAPI stains and medullary voids determined with the use of an ImageJ script and defined as spaces within the medulla lacking in K5 stain and > 5000 µm^2^ in area. Scale bar 100 µm of each main image and 25 µm of zoom region. (**b**) Mean number of voids and (**c**) mean void area per mouse. Microscopy images acquired with Zen 2012 (ZEISS ZEN Microscope Software for Microscope Components) and processed with FIJI/ImageJ (Fiji (imagej.net)) and figure compiled with Microsoft PowerPoint (Microsoft PowerPoint Slide Presentation Software|Microsoft 365). Symbols represent mean from 3–4 images per mouse, n = 2–4 mice per group, combined from 2 independent experiments, mean ± SEM.

A recent study showed that emigration of mature thymocytes from the thymus was dependent on the type-2 cytokine receptor IL-4Rα chain^37^. Lack of IL-4Rα and impaired thymic emigration led to accumulation of mature thymocytes in the medulla around perivascular spaces, leading to the formation of large medullary areas devoid of epithelial stromal cells. Moreover, this study also showed the appearance of these medullary epithelial cell-free areas in CD1d-deficient mice, suggesting a key role for NKT cells as a source of IL-4 that regulates thymic emigration^37^. CD1d-deficient mice lack both type I and type II NKT cells, both of which can produce IL-4 and IL-13. Therefore, we sought to explore if these epithelial cell-free areas (that we refer to as voids) were specifically due to the lack of type I NKT cells by comparing medullary thymic epithelial cell staining in WT, CD1d^−/−^ and TCR Jα 18^−/−^ thymuses (the latter deficient in type I but not type II NKT cells). While we were able to detect some areas in the medulla that were devoid of thymic epithelial cells (as outlined with green line) (Fig. 4a), these did not resemble the clear ring like structures that were previously reported^37^. Moreover, we found no significant difference in the number of voids in CD1d^−/−^ (Fig. 4b) and while the average size of these was marginally increased in CD1d^−/−^ mice, this was also not significant (Fig. 4c). We also failed to detect any increase in the size of medullary voids in type I NKT cell deficient Jα18^−/−^ mice. As these data were acquired in B6 strain background, we also carried out similar studies in BALB/c mice where NKT cells produce higher levels of IL-4, but again, we were unable to detect a clear increase in the appearance of medullary voids (Supplementary Fig. S3). As a positive control for detection of medullary epithelial cell voids, we also tested thymuses from NOD mice, as these are known to contain large numbers of B cells in the thymic medulla that lead to enlarged perivascular spaces that disrupt thymic epithelial structure^38^. Clear evidence of medullary voids were detected in NOD mouse thymi (Fig. 4a).

Another consideration was that CD1d-deficient mice have increased MAIT cells, particularly on the BALB/c background^39^. Therefore, we also examined Vα19 TCR transgenic.Cα^−/−^ mice (Vα19Tg) and MR1^−/−^ mice, which have increased, and decreased, MAIT cells respectively^40,41^ for medullary voids, but these were also not significantly different from control B6 mice (Supplementary Fig. S3). Taken together, while we have confirmed the presence of NKT cells in the thymic medulla, we have been unable to verify that the absence of any thymic NKT cell population has an impact on thymic medullary architecture.

### In situ observation of NKT cells in peripheral organs

As well as primary and secondary lymphoid organs, NKT cells reside in a range of non-lymphoid organs, particularly in the liver, small intestine and lungs. Thus, we endeavoured to detect NKT cells in a range of peripheral organs using CD1d tetramer staining. While CD1d-α-GalCer tetramer^+^ cells were detected within the small intestine, lung, kidney and heart of BALB/c WT mice (Supplementary Figs. S4–S7), many of these stained brightly with the tetramer, but not with CD3. Furthermore, the importance of the unloaded CD1d tetramer control stain was highlighted by the existence of CD3^−^ cells that bound to unloaded CD1d tetramer in these tissues. For example, in the small intestine non-specific (CD1d tetramer positive, but CD3 negative) staining could be observed prominently in the villi, regardless of whether CD1d was loaded with negative control antigen (Supplementary Fig. S4a) or α-GalCer (Supplementary Fig. S4b). Similarly, in the lung (Supplementary Fig. S5), kidney (Supplementary Fig. S6) and heart (Supplementary Fig. S7), GD3 and α-GalCer-loaded CD1d tetramer positive, but CD3 negative staining could be also observed. Importantly, despite these non-specifically stained cells, CD3^+^CD1d-α-GalCer tetramer^+^ cells were also detected, albeit infrequently, in Payer’s Patch (Supplementary Fig. S4b) and villi (Supplementary Fig. S4c) of small intestine. Rare NKT cells were detected in lungs within the alveolar ducts (Supplementary Fig. S5b). In kidney, NKT cells could be observed between convoluted tubules (Supplementary Fig. S6b) and in heart within the myocardium between perimysial septa (Supplementary Fig. S7b). Because NKT cells were so infrequent in lung, kidney and heart (1–4 per section), it was difficult to determine their preferred location.

### Relocation of NKT in the spleen following α-GalCer stimulation

**Figure 5:**
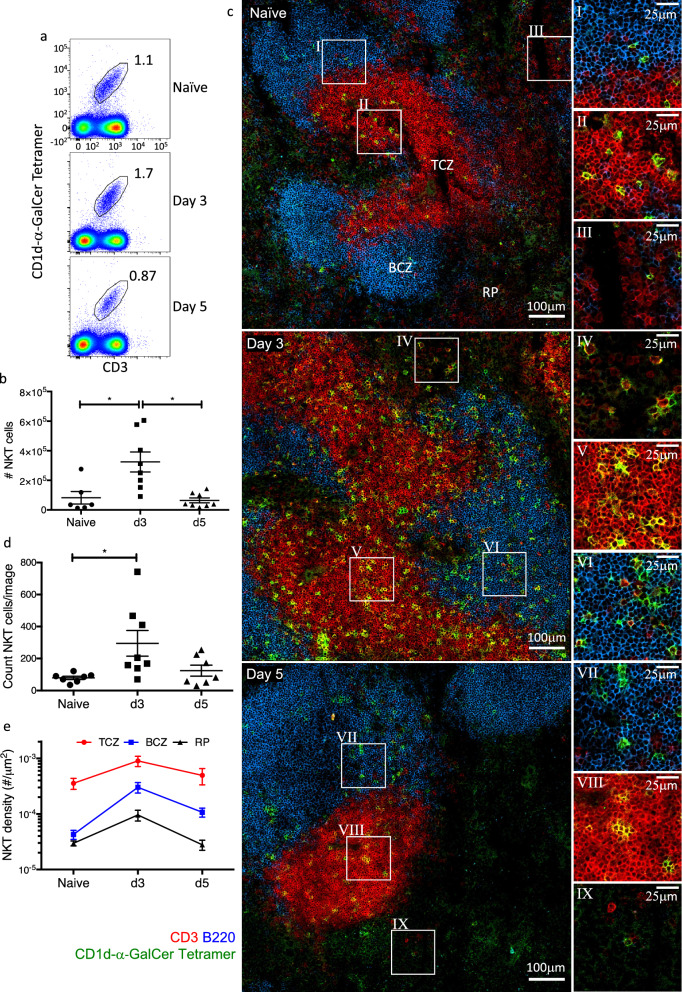
The expansion and redistribution of NKT cells following in vivo stimulation with α-GalCer. BALB/c WT mice were injected intraperitoneally with 2 μg of α-GalCer or left unstimulated. On days 3 and 5 post injection the spleens were harvested. Half of the spleen of each mouse were prepared for FACS analysis and stained with anti-mouse B220 and CD3 antibodies and CD1d-α-GalCer tetramer. The matched half of the spleens were snap frozen and cut into 7 μm sections and stained with B220 and CD3 antibodies and CD1d-α-GalCer tetramer for histological analysis. (**a**) Representative FACS plots of splenic CD3^+^CD1d-α-GalCer tetramer^+^ cells for each time point and (**b**) mean number of NKT cells ± SEM. Cells gated on live B220^−^ lymphocytes. (**c**) Representative image showing CD1d-α-GalCer tetramer (green), B220 (blue) and CD3 (red) staining within the splenic T cells zone (TCZ), B cells zone (BCZ) and red pulp (RP), scale bar 100 μm, with corresponding zoomed regions of interest, scale bar 25 μm. (**d**) Total count of NKT cells per 0.63 mm^2^ image and (**e**) density of NKT cells within each splenic region at each time point. Microscopy images acquired with Zen 2012 (ZEISS ZEN Microscope Software for Microscope Components) and processed with FIJI/ImageJ (Fiji (imagej.net)) and Imaris 9.3 (Microscopy Image Analysis Software—Imaris—Oxford Instruments (oxinst.com)) and figure compiled with Microsoft PowerPoint (Microsoft PowerPoint Slide Presentation Software|Microsoft 365). Mean ± SEM. Symbols represent each mouse of n = 7–8 mice per group combined from 2 independent experiments, Kruskal–Wallis with Dunn’s post-hoc test, * = *p* < 0.05.

Having demonstrated the in situ location of NKT cells in lymphoid tissues, we next examined what happened to the location of these cells following in vivo activation. Mice were injected intraperitoneally with 2 μg α-GalCer and changes to the NKT cell population were determined on days 3 and 5 post injection. Previous work has shown an increase in the NKT cell population following activation by endogenous α-GalCer^42–44^. As expected, FACS analysis of spleen cell suspensions from these mice showed a rapid increase in NKT cell numbers in the spleen 3 days post injection, which decreased by day 5 post injection (Fig. 5a, b). This was also reflected by a clear increase in CD1d-α-GalCer tetramer staining of spleen tissue sections taken at day 3 after activation, and subsequent reduction in the extent of this staining at day 5 (Fig. 5c, d). It is unclear whether this increase in splenic NKT cells results from local intra-splenic expansion, or recruitment from other tissues, or a combination of the two. As expected, CD1d-α-GalCer staining was associated with CD3 staining (Supplementary Fig. S8). As previously shown, CD3^+^CD1d-α-GalCer^+^ NKT cells in unstimulated mice were observed throughout the spleen with largely equal proportions of NKT cells in the BCZ and RP, while most (~ 10 × as many) were located within the splenic TCZ. By day 3 following antigen stimulation, the proportion of NKT cells in all three regions increased, and some redistribution of NKT cells was apparent by day 5 (Fig. 5e). While NKT cells within the TCZ remained the largest population, increasing ~ 2.5-fold 3 days post stimulation, the BCZ saw the greatest increase in NKT cell density such that by day 3, the population of NKT cells in this region had increased ~ 7-fold. We also observed a ~ 3-fold increase in the frequency of NKT cells within the RP 3 days following stimulation. By day 5, the frequency of CD3^+^CD1d-α-GalCer^+^ NKT cells in the TCZ and RP had declined to levels that were similar to unstimulated mice. In contrast, the frequency of cells in the BCZ remained ~ 2.8-fold higher compared to unstimulated mice. The increased CD3^+^CD1d-α-GalCer^+^ NKT cells within the BCZ may reflect their role in modulating B cell responses following activation.

We next investigated NKT cells in the periphery following stimulation. The liver is a major non-lymphoid organ known to contain a large population of NKT cells. Following injection of α-GalCer, an increased population of NKT cells was detected that followed a similar pattern as in the spleen; a large increase 3 days post stimulation, which had contracted by day 5 (Supplementary Fig. S9a and b). Detecting NKT cells in situ in this organ proved challenging due to its large size, and low frequency of lymphocytes in general; nonetheless, NKT cells were detected, albeit infrequently, within the liver sinusoids. Consistent with the cytometry data, NKT cells were more frequent at day 3 where it was possible to observed 2–7 NKT cells per field of view. In livers of either unstimulated or day 5 post challenge mice, however, they remained difficult to locate (1–3 per section). Importantly, regardless of the time point, these cells co-stained with CD3 and this staining was not observed in negative control sections (Supplementary Fig. S9c).

### Multiparameter histo-cytometry of splenic NKT cells, T cells, B cells and antigen-presenting cells

**Figure 6:**
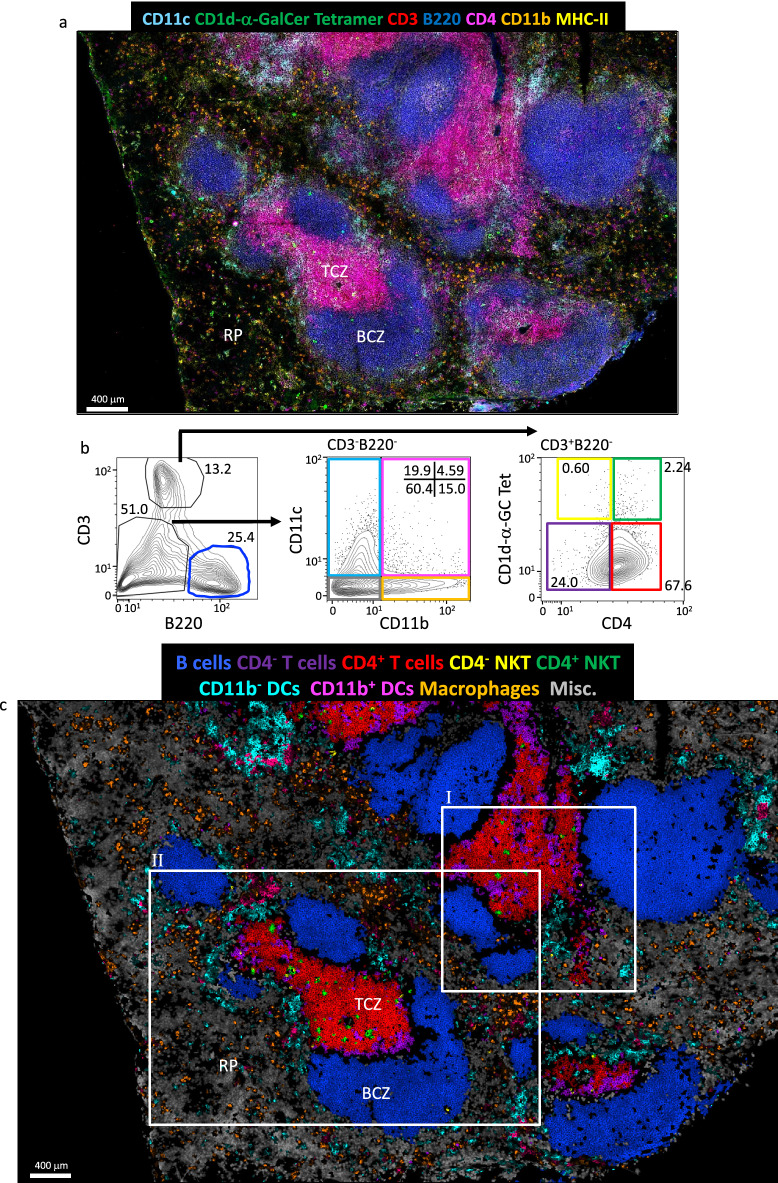

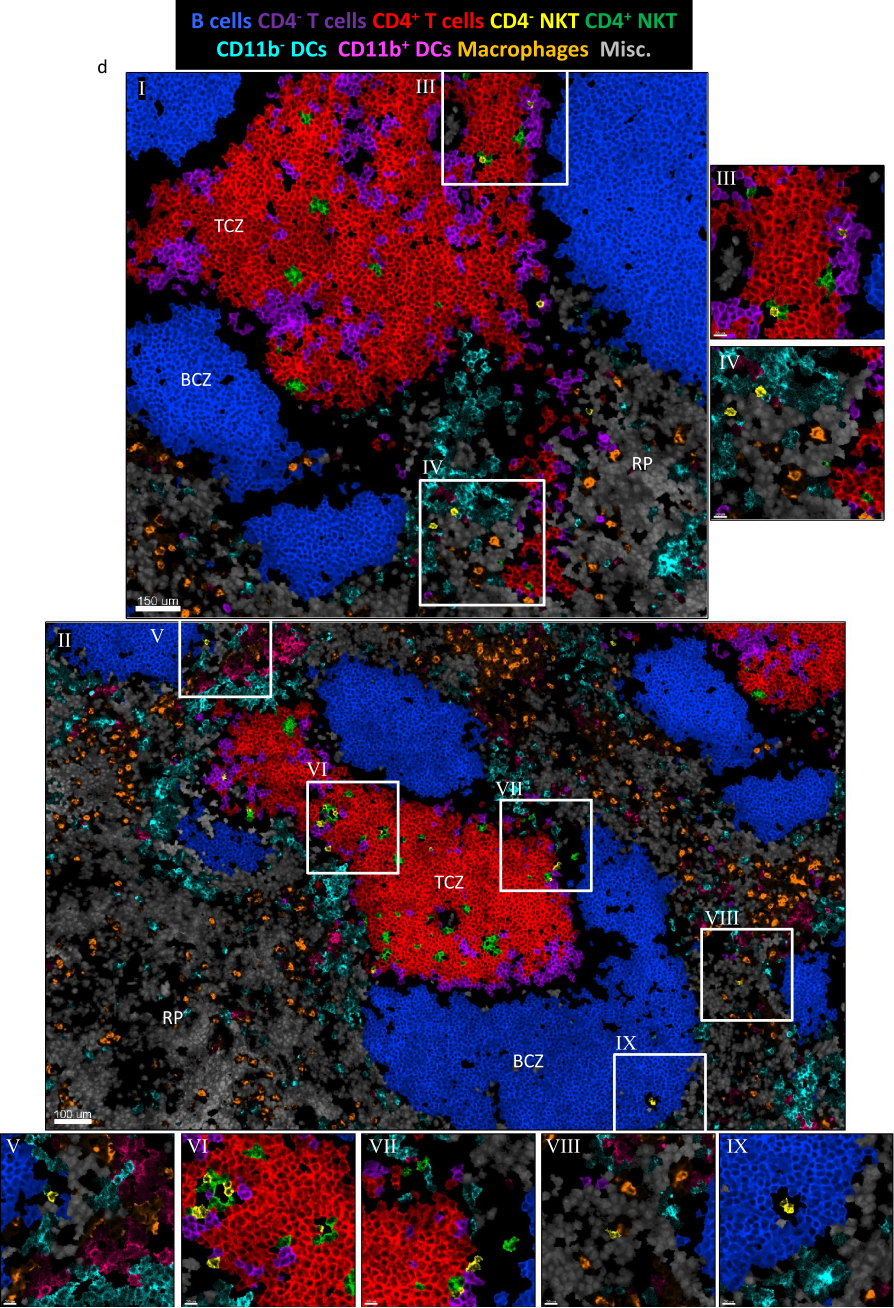
Multiparameter in situ analysis of NKT cells and antigen-presenting cells. Spleen sections from BALB/c WT mice were cut and stained with anti-mouse B220, CD3, CD4, CD11b, CD11c, MHC-II antibodies, CD1d-α-GalCer tetramer followed by DAPI counterstain. Following spectral imaging, linear unmixing and deconvolution was performed followed by conventional compensation. (**a**) Representative image of the resulting separated channels CD11c (cyan), CD1d-α-GalCer (green), CD3 (red), B220 (blue), CD4 (purple), CD11b (orange) and MHC-II (yellow). Single cell masks were made based on the DAPI stain and staining data of the segmented cells exported for histo-cytometry analysis. (**b**) Representative histo-cytometry plots used to identify B cells (blue), CD11b^−^ DCs (cyan), CD11b^+^ DCs (pink), macrophages (orange), CD4^−^ NKT cells (yellow), CD4^+^ NKT cells (green), CD4^−^ T cells (purple), CD4 + T cells (red) and miscellaneous (misc) cells (CD3^−^B220^−^CD11c^−^CD11b^−^, gray). Numbers show percentage of cells with gates of total cells in the plot. (**c**) Representative image of the cells as defined by histo-cytometry masked back to the original locations of those cells and (**d**) zoom regions. Cells masked with the original stains. Scale bar of image in A and C 400 μm, I and II 150 μm and III to IX 30 μm. Splenic Red Pulp (RP), B cell zone (BCZ) and T cell zone (TCZ). Microscopy images acquired with Zen 2012 (ZEISS ZEN Microscope Software for Microscope Components) and processed with FIJI/ImageJ (Fiji (imagej.net)), Imaris 9.3 (Microscopy Image Analysis Software—Imaris—Oxford Instruments (oxinst.com)), Huygens 20.04 (Huygens Software|Scientific Volume Imaging (svi.nl)) and FlowJo 10 (Home|FlowJo, LLC) and figure compiled with Microsoft PowerPoint (Microsoft PowerPoint Slide Presentation Software | Microsoft 365). One mouse representative of 3 independent mice/experiments.

The cellular environment within lymphoid organs is complex and involves many different subsets of immune cells influencing each other. Indeed, NKT cells interact with and modulate the responses of a range of innate and adaptive immune cells^36^. These varying cell types are difficult to investigate together with typical histological techniques due to lack of methods to stain for the multitude of cell surface receptors required to fully identify the diverse array of immune cells in situ. Therefore, we used multiparameter histo-cytometry^45^ to visualise NKT cells in the context of other immune cells. To this end, spleen sections were stained with a range of cell surface receptors (CD11c, CD11b, CD3, CD4, CD1d-α-GalCer, B220 and MHC-II) and cell nuclei with DAPI, and the separate fluorescence of each stain was determined by spectral unmixing and compensation (Fig. 6a and Supplementary Fig. S10a). Cells were segmented based on the DAPI stain (Supplementary Fig. S10b) and separate cell populations determined by conventional cytometry analysis (Fig. 6b). Clear populations of B cells and T cells as well as NKT cells and various APC populations could be identified and were plotted back to cells, masked to their original location within the image (Fig. 6c). CD4 positive and negative T and NKT cells proved difficult to separate using this technique, likely owing to them being closely packed and intermingled in the similar spatial location, namely the TCZ. Overall, however, the various cell populations could be mapped within the same histological regions within the spleen as they were observed in the original stained sample (Supplementary Fig. S10d), but with greater clarity, providing a better way to analyse these cells in association with other diverse cell types. This approach supports our previous observations that NKT cells were largely located within the TCZ, of which most were CD4^+^ (Fig. 6d, zoom panels III, VI and VII), while a small number of CD4^−^ NKT cells were found in the BCZ (Fig. 6d, zoom panels V and IX) and RP (Fig. 6d, zoom panels IV and VIII). Some NKT cells were also found in marginal zone (Fig. 6d, zoom panels V and VII). As expected, the majority of APCs were observed outside the white pulp, with macrophages prominent throughout the RP, while CD11b^−^ and CD11b^+^ DCs mostly associated with the marginal zone (Fig. 6d, zoom panels IV, V, VII and VIII). In particular, CD4^−^ NKT cells were observed in amongst the CD11b^−^ DCs (Fig. 6d zoom panel IV and V).

### In situ identification of MAIT cells with MR1-5-OP-RU tetramers

**Figure 7:**
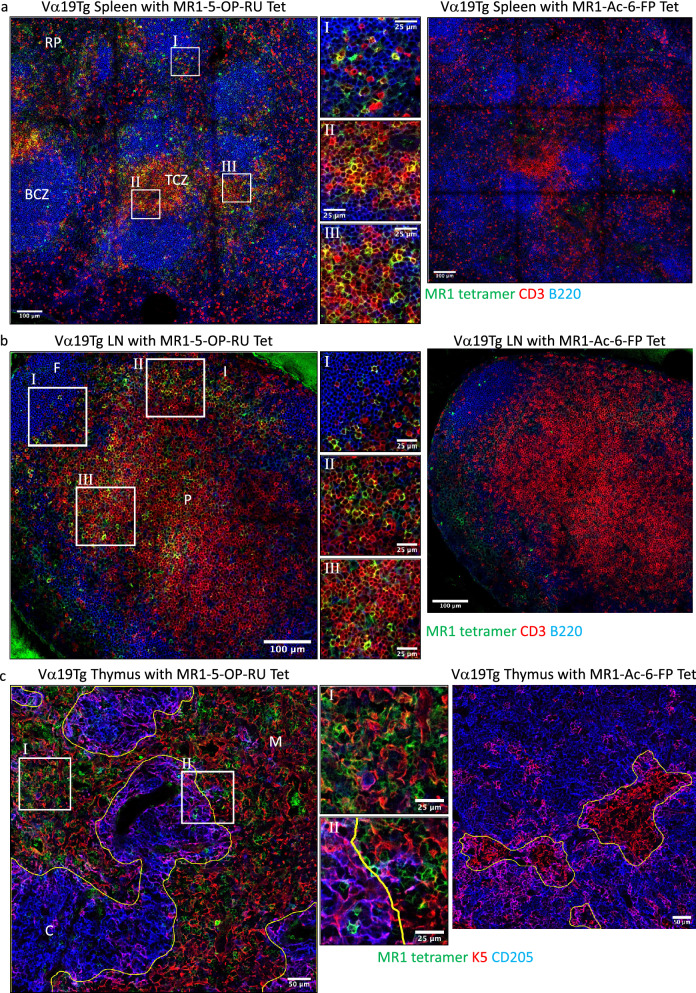
Identification of MAIT cells in Vα19 TCR transgenic mice in situ. Sections from Vα19Tg.Cα^−/−^ mice spleen (**a**), LN (**b**) and thymus (**c**) were stained with MR1 tetramers loaded with 5-OP-RU or Ac-6-FP and anti-CD3 and anti-B220 antibodies or anti-CD205 and cytokeratin-5 (K5). Representative images of spleen and LN showing staining with MR1 tetramer (green), CD3 (red) and B220 (blue), and thymus with cytokeratin-5 (red) and CD205 (blue), scale bar 100 μm. Zoomed regions scale bar 25 μm. Splenic red pulp (RP), B cell zone (BCZ) and T cell zone (TCZ). LN medulla (M), follicle (F) and paracortex (P). Thymic cortex (C) and medulla (M). Microscopy images acquired with Zen 2012 (ZEISS ZEN Microscope Software for Microscope Components) and processed with FIJI/ImageJ (Fiji (imagej.net)), Imaris 9.3 (Microscopy Image Analysis Software—Imaris—Oxford Instruments (oxinst.com)) and figure compiled with Microsoft PowerPoint (Microsoft PowerPoint Slide Presentation Software|Microsoft 365). Data representative of three independent experiments, Tet = Tetramer.

Very little is known about the location of MAIT cells within tissues. Similar to the technique to stain for NKT cells, MR1 tetramers loaded with MAIT cell-specific antigen 5-OP-RU were used to identify MAIT cells. MAIT cells are much rarer than NKT cells in mice^41^ so to begin with, we used Vα19Tg mice, which contain a much larger proportion of MR1 tetramer-binding T cells than WT mice. A clear population of brightly stained MR1-5-OP-RU tetramer^+^ CD3^+^ T cells was observed in spleen, lymph nodes and thymus of Vα19Tg mice. Importantly, this staining was not evident on sections stained with control acetyl-6-formylpterin (Ac-6-FP)-loaded MR1 tetramer (Fig. 7), supporting the specificity of this staining for MR1-5-OP-RU reactive T cells. In all organs, the pattern of MR1-5-OP-RU tetramer staining in the Vα19Tg mice was similar to that observed for NKT in WT mice, with the major difference being the greater number of MR1 tetramer^+^ cells observed in the Vα19Tg mice. In spleen, these MR1 tetramer^+^ cells were mainly detected in the TCZ and less frequent cells were detected in the BCZ and RP (Fig. 7a). Similarly, MR1 tetramer^+^ cells were prominent in the LN paracortex, though unlike NKT cells, they were also observed in the Vα19Tg LN medulla (Fig. 7b). Curiously, non-specific staining in the region of the glass slide where the tissue embedding medium (Optimal Cutting Temperature, OCT) was located was consistently observed with the 5-OP-RU-loaded MR1 tetramer, but not MR1-Ac-6-FP (Figs. 7b and S13a). The reason for this effect is unknown but may be a result of the natural fluorescence of 5-OP-RU^46^. In the thymus of Vα19Tg mice, the vast majority of MR1 tetramer^+^ cells were detected in the thymic medulla (Fig. 7c). Together these results show that, similar to the identification of NKT cells with CD1d-α-GalCer tetramers, MR1-5-OP-RU tetramers can specifically stain MR1-restricted cells in situ.

**Figure 8:**
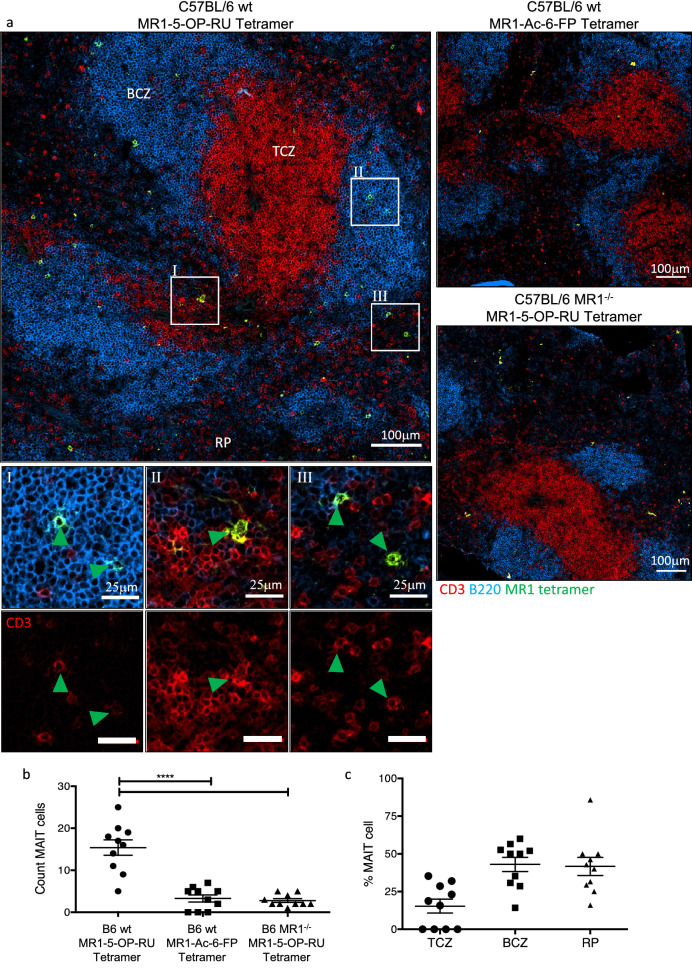
Identification of MAIT cells within WT mouse spleen in situ. 7 μm sections of spleens from B6 WT or B6.MR1^−/−^ mice stained with MR1 tetramers loaded with 5-OP-RU or Ac-6-FP and anti-B220 and CD3 antibodies. (**a**) Representative images showing MR1 tetramer (green), B220 (blue) and CD3 (red) staining within splenic red pulp (RP), B cell zone (BCZ) and T cell zone (TCZ) of the splenic white pulp, scale bar 100 μm with corresponding zoomed regions of interest as shown, scale bar 25 μm. (**b**) Count of MR1-5-OP-RU tetramer^+^ CD3^+^ MAIT cells per 0.63 mm^2^ image and **c** proportion of MAIT cells within each splenic region of the total number of NKT cells per image. Microscopy images acquired with Zen 2012 (ZEISS ZEN Microscope Software for Microscope Components) and processed with FIJI/ImageJ (Fiji (imagej.net)) and Imaris 9.3 (Microscopy Image Analysis Software—Imaris—Oxford Instruments (oxinst.com)) and figure compiled with Microsoft PowerPoint (Microsoft PowerPoint Slide Presentation Software|Microsoft 365). Mean ± SEM, symbols represent each mouse of n = 10 mice combined from 2 independent experiments, one-way ANOVA with Dunnett’s post-hoc test, **** = *p* < 0.001.

Next, we examined the location of MAIT cells in non-TCR transgenic B6, BALB/c and B6-MAIT_CAST_ (CAST) mice (Fig. 8 and Supplementary Fig. S11). The latter were also tested because this congenic strain is reported to have an increased number of MAIT cells, due to increased intrathymic selection of these cells^47^. While infrequent, nonetheless, MAIT cells could be readily observed in spleens stained with MR1-5-OP-RU tetramer in all three mouse strains. Importantly, these cells were not detected in the spleens from B6, BALB/c or CAST mice when stained with MR1-Ac-6-FP tetramer, nor were they detected in B6.MR1^−/−^ or CAST.MR1^−/−^ mice stained with MR1-5-OP-RU tetramer (Figs. 8b, S11b and e). Furthermore, MR1-5-OP-RU staining associated with CD3 staining. Similar numbers of MAIT cells (~ 10–20 cells) were observed in the spleens of B6 and CAST mice, while in BALB/c spleen only 5 cells on average could be observed. Interestingly, and in contrast to MAIT cells in the Vα19Tg mice, for each of the non-transgenic strains tested, most of the MAIT cells were detected outside the TCZ; collectively within the BCZ and RP of the spleens (Figs. 8c and S11c and f). We also attempted to investigate the locational differences between the NKT cells and MAIT cells by co-staining spleen sections of B6 and BALB/c mice with both CD1d-α-GalCer and MR1-5-OP-RU tetramers (Supplementary Fig. S12a and b). A challenge with this approach is that both tetramers work optimally using the same fluorochrome, so a suboptimal fluorochrome was used for CD1d-α-GalCer tetramer staining. Furthermore, MAIT cells were more readily detectable in B6 mice while NKT cells were more detectable in BALB/c mice, so both were tested. Nonetheless, this co-staining supported the concept that NKT cells and MAIT cells occupy different locations in spleen. The thymuses of all three strains of mice were also stained with MR1-5-OP-RU tetramer (Supplementary Fig. S13a–c). While a very small number of MR1-5-OP-RU^+^CD3^+^ cells could be seen in the cortex of the thymus of B6 and CAST mice, they were undetectable in BALB/c mice. This is not surprising because we have previously published that MAIT cells are exceedingly rare in mouse thymus^41^. Furthermore, a similar number of MR1 tetramer^+^ but CD3^−^ cells were also observed in negative controls, which again highlights the importance of appropriate controls in attempting to detect MAIT cells in situ. Nevertheless, with appropriate caution based on detection of very few cells, this suggests that MAIT cells may preferentially reside in the cortex of the thymus of non-Vα19 TCR transgenic mice, in contrast to their medullary location in Vα19 TCR transgenic mice (Fig. 7c).

The distribution of MAIT cells following in vivo activation was investigated by intranasal (i.n.) administration of 5-OP-RU in CAST mice and MR1-5-OP-RU tetramer staining of mediastinal LN (Supplementary Fig. S14). Similar to the MR1 tetramer staining in the spleen, while only a small number of MAIT cells could be observed in these LNs, these were not detected when the same tissue was stained with negative control Ac-6-FP-loaded MR1 tetramer. Notwithstanding the small numbers of MAIT cells detected, there appeared to be an increase in their abundance following 5-OP-RU challenge (Supplementary Fig. S14c) and most were located within or on the edge of the paracortex (Supplementary Fig. S14b and d).

While interpretation of these data is limited by the scarcity of cells detected in non-TCR transgenic mice, these results suggest that MAIT cells in WT mice occupy specific locations within lymphoid tissues, primarily located in regions of the spleen that differ from NKT cells and conventional T cells and that this is not reflected by the location of MAIT cells in TCR transgenic mice.

## Discussion

This report examines the location of both NKT cells and MAIT cells in situ using CD1d-α-GalCer and MR1-5-OP-RU tetramers, respectively. These reagents are widely used in flow cytometry, but very few studies have employed them for in situ detection of NKT and MAIT cells. We demonstrate the feasibility of using these reagents to identify NKT and MAIT cells in situ. While the tetramers we used were produced in house, previous studies have used CD1d tetramers made in house or sourced externally to detect NKT cells in situ^16–20^. Provided high quality tetramers are used, the techniques described here should be broadly amendable regardless of the tetramer source. We also emphasise the need to include appropriate controls, such as GD3-loaded CD1d tetramers; Ac-6-FP-loaded MR1 tetramers; and CD1d and MR1 deficient mice, to ensure specific detection of NKT cells and MAIT cells respectively. Using these controls, we identified false-positive staining that was sometimes similar to, or more abundant than, the actual NKT and MAIT cell staining. The basis for this false positive staining is unknown, but it can also be observed by flow cytometry^41,48^ where tetramers can stain non-T cells. In these flow cytometry settings, co-labelling of CD1 and MR1 tetramers is usually accompanied at least by anti-TCR or anti-CD3 antibodies to ensure that T cells are being analysed.

Previous attempts at in situ tetramer staining showed how challenging this approach is. Indeed, the first reported use of CD1d-α-GalCer tetramer in situ successfully stained NKT cells in Vα14 TCR transgenic mice where these cells are abundant, but in that study, NKT cells could not accurately be detected in non-transgenic mice^16^. Within the Vα14 transgenic mice, NKT cells were observed in the TCZ of the spleen and lymph nodes. More recently, some studies have succeeded in using CD1d-α-GalCer tetramers to identify NKT cells in non-transgenic mice^17–20^, although as we demonstrate in this study, non-specific CD1d-tetramer staining may complicate interpretation. In our hands, this non-specific staining was typically associated within both the RP and BCZ of the spleen. Therefore, without specifically defining NKT cells with tetramer and CD3 or TCR staining, it is possible that in some previous studies, non-specific staining may have given the impression that a higher proportion of NKT cells exist in the BCZ and RP. Furthermore, the varying methods of tetramer staining themselves have the potential to influence results. Previous investigations using CD1d tetramer have taken a whole tissue staining approach^18–20^ where whole segments of tissue are immersed in the stain and the tetramer diffuses passively into the specimen. A possible problem with this approach could be uneven staining where tetramer penetration may be limited as has been seen for some antibodies in techniques that stain whole tissue segments^49^. In contrast, we have stained thin tissue sections directly with the tetramer, ensuring that NKT cells are evenly stained across all regions of the section.

Early investigations into the location of NKT cells in the spleen and lymph node have relied on staining for surrogate markers such as NK1.1 or CXCR6-GFP reporter, sometimes combined with adoptive transfer of labelled NKT cells^8,10,12,13^. These studies point towards the lymph node medulla and splenic RP and MZ as the location of the majority of NKT cells in these organs. However, neither of these approaches exclusively label NKT cells, and furthermore, not all NKT cells express NK1.1 or CXCR6^8,10^. Therefore, many of the cells identified might not be NKT cells and some NKT cells would likely be missed from the analysis. Furthermore, adoptive cell transfer may affect the locations that NKT cells traffic to post transfer, particularly if they are activated by the transfer process. Indeed, in contrast to studies using NKT cell transfer, the first studies to use in situ CD1d-α-GalCer tetramer staining primarily located resting NKT cells in the splenic TCZ^16,17^. Using a CD1d-α-GalCer tetramer-based approach, we investigated the location of NKT cells in the spleens of BALB/c and B6 mice. While acknowledging the ages of the mice used in the study may impact on the numbers of innate-like T cells^56^, there is no reason to suggest that this will impact on their intra-organ distribution. The strain-specific differences we observed, where most NKT cells were localised in the TCZ, and occasionally in the B cell follicles of the WP in BALB/c mice, versus a RP location in B6 mice, is in line with a previous study^18^. In the spleens of both mouse strains, NKT1 cells were localised mostly outside the splenic WP, while most NKT2 cells were found within the WP^18^. Of the NKT subsets, NKT2 cells are more abundant in BALB/c mice^50^ and have a greater expression of CCR7^51,52^. Together, this may explain the greater abundance of NKT cells in the splenic WP of BALB/c than B6 mice^50^. This may also explain our finding by colocalization analysis and histo-cytometry that CD4^+^ NKT cells were more frequent in the TCZ, because most NKT2 cells express CD4 whereas NKT1 cells can be CD4^+^ or CD4^−^^50,53^.

While NKT cells were mainly in the TCZ rather than the BCZ of unstimulated BALB/c mice, a large increase in BCZ NKT cells was observed 3 days following α-GalCer mediated in vivo NKT cell activation. NKT cells are well known to provide B cell help through germinal centre formation, antibody class switching and affinity maturation^19,54–56^. Depending on the infection and mode of activation, NKT cells can provide both cognate and non-cognate help to B cells. For instance, NKT activated in response to α-GalCer modulate B cell response in a CD1d-TCR dependent manner^54–56^, while during viral infection, activated NKT cells can influence B cell responses in a non-cognate fashion via the production of cytokines such as IL-4^19^. Previously, tetramer staining has been used to investigate NKT cell non-cognate B cell help during influenza virus infection^19^. It was seen that early in infection NKT cells migrated to the B cell border in the mediastinal LN and provided non-cognate B cell help via the local production of IL-4. Similarly, within 4 h following intravenous antigen injection, CD1d tetramer staining showed that NKT cells rapidly migrate to the splenic MZ where they interact with MZ DCs and express IL-4^17^. Similarly, we saw the expansion and change in the location of NKT cells in the spleen following activation, though in contrast, by day 3 following α-GalCer injection, we observed that a large proportion of NKT cells resided in the BCZ. Given that B cells are capable of presenting α-GalCer to NKT cells^54,56^. It is possible that following early migration to the MZ, NKT cells then migrate further into the BCZ where cognate B cell-NKT interactions occur. It is likely that in a situation involving glycolipid and TCR-dependent activation of NKT cells, NKT cells enter the BCZ to provide cognate B cell help directly in the germinal centre.

We observed that the majority of NKT cells are located within the medulla of the thymus. This aligns with a previous study of thymic NKT cells in either B6 or BALB/c mice^18^. It is known that an interdependency exists between medullary thymic epithelial cells (mTEC) and thymocyte development^57^, including a key role for NKT cells via RANKL and IL-13R^37,58^. Furthermore, in the absence of NKT cells in CD1d^−/−^, thymocyte emigration was impaired, leading to areas of the medulla where mature thymocytes accumulated, giving the appearance of epithelial cell-free voids by immunohistology^37^. We intended to investigate whether Jα18^−/−^ mice, which lack type I but retain type II NKT cells, also developed medullary voids, which would discriminate between the role of these two NKT cell subsets in regulating thymic emigration. However, we were unable to detect an increase in these voids in either CD1d^−/−^ or Jα18^−/−^ thymuses, on either B6 or BALB/c strain backgrounds. It is unclear why our observations differ from those previously published but given the influence of gut microbiota on NKT cells^59–61^ it is possible that differences in gut microbiome of our mice compared to those used in the previous study may be responsible. Further studies will be required to elucidate the apparent conflict between these data reported here and those reported previously.

We have also demonstrated the in situ localisation of MAIT cells in spleen and lymph node with MR1-5-OP-RU tetramer and CD3 co-staining. Because MAIT cells are very rare in mice^41^ we first used Vα19 TCR transgenic mice where these cells are more abundant and easier to detect. The use of transgenic mice is not without its limitations. For example, the expression of transgene and flowthrough effects on development and expression of PLZF^41^ requires caution when interpreting the location of the MR1 tetramer^+^ cells within the Vα19Tg mice. Nevertheless, the data from Vα19Tg mice reassured us that we were indeed able to detect MR1 tetramer^+^ T cells in situ. Though the scarcity of MAIT cells in non-transgenic mice remains an obstacle to their study, our analysis of many sections from many mice and use of controls such as Ac-6-FP-loaded MR1 tetramer and MR1^−/−^ mice, allowed us to determine that MAIT cells appear to occupy different locations to NKT cells. For example, in contrast to NKT cells, in non-TCR transgenic mice, most MAIT cells were located outside the TCZ in spleen, which may result from differential expression of chemokine receptors. It is known that MAIT cells express CXCR6 and a subset expresses CCR9, which correlates with their well-known association with mucosal tissues, but not the TCZ homing chemokine CCR7^41,62^. Conversely, of the NKT subsets, NKT2 cells have a greater expression of CCR7^51,52^. As such, it is possible that the MAIT cells detected in the spleen are simply passing through the organ to other peripheral organs, while NKT2 cells are directed to TCZ. Alternatively, it is possible that MAIT cells are fulfilling a function in the RP and that the majority MAIT cells are expressing similar chemokine receptors as NKT1 cells and as such are directed to similar locations. The RP provides an environment to quickly scan for blood borne antigens^63^, which may place RP-MAIT cells in a prime position to quickly respond to these antigens. Indeed, MAIT cells are important in the control of many bacterial infections (reviewed in^5^) including the control of blood-borne infections such as *E. coli* and *M. absceccus*^64^. MAIT cells were also clearly observed in the thymus, mostly within the medulla but occasionally in the cortex, of Vα19 transgenic mice, thus similar to NKT cells in their distribution. However, as mentioned, caution will need to be applied when interpreting results from Vα19Tg mice. Furthermore, while rare examples of MR1 tetramer staining of CD3^+^ cells were also seen in the thymic cortex of wildtype mice, we remain cautious about overinterpreting these data because there were also rare instances of MR1 tetramer staining in negative controls on the non-Vα19 transgenic mice. These may reflect rare T cells that express a TCR with the ability to bind to MR1, even in the absence of MR1-dependent intrathymic selection.

Taken together, this study demonstrates the use of CD1d and MR1 tetramers to detect NKT and MAIT cells in situ, respectively, and highlights the power of this technique when used in conjunction with TCR co-stains as well as appropriate tetramer and tissue controls. This approach should provide valuable insight into the function of these cells in different immunological and disease related settings.

## Methods

### Animal models

BALB/c WT, BALB/c.Jα18^−/−^, BALB/c.CD1d^−/−^, B6 WT, B6.Jα18^−/−^, B6.CD1d^−/−^, B6.MR1^−/−^, Vα19Tg and NOD were housed in Peter Doherty Institute Biological Research Facility (PDI BRF, Melbourne, Victoria, Australia) and either purchased from the PDI BRF or Animal Resource Centre (Perth, Western Australia, Australia). B6-MAIT_CAST_ WT and B6-MAIT_CAST_.MR1^−/−^ (kindly supplied by Olivier Lantz, Institut Curie, Paris France) were housed in the Peter MacCallum Cancer Centre animal facility. Male or female mice 6–12 weeks of age, matched to age and sex, were housed and experiments performed in accordance with the relevant guidelines and regulations and under approval of the University of Melbourne Animal Ethics Committee (ethics number 1914739) or Peter MacCallum Cancer Centre animal ethics committee (ethics number E582). All experiments were carried out in compliance with the ARRIVE guidelines.

### Chemical synthesis of MAIT cell antigen

5-OP-RU (5-(2-oxopropylideneamino)-6-d-ribitylaminouracil) was synthesised as previously detailed^65^, by combining 5-A-RU (5-amino-6-d-ribitylaminouracil) and methylglyoxal in DMSO-d_6_, where it is very stable.

### Tetramer production

Recombinant mouse CD1d tetramers were generated as previously described^66^. Purified mouse CD1d-biotin was incubated with PBS-44 C24:1 α-GalCer analogue (a gift from P. Savage, Brigham Young University, Provo, UT) or GD3 (Matreya LLC) at a 1:6 (CD1d:ligand) molar ratio overnight at room temperature. Mouse MR1-5-OP-RU and MR1-Ac-6-FP tetramers were generated from purified recombinant mouse MR1 monomers as previously described^3,67^. The loaded CD1d or MR1 monomers were tetramerised with the sequential addition (1/5 total required volume) of unlabelled streptavidin, streptavidin-PE (BD) or streptavidin-FITC (BD) over a series of 10 min incubations at 4 °C for a total of 50 min.

### In vivo antigen stimulation

To stimulate NKT cells, male BALB/c mice 6–8 weeks of age were injected intraperitoneally with 2 μg α-GalCer (KRN7000, C26:0, Alexis Biochemicals) or with PBS alone. On days 3 and 5 post infection the mice were killed by CO_2_ asphyxia, liver was perfused with PBS via the portal vein and livers and spleens were removed.

To stimulate MAIT cells, B6-MAIT_CAST_ mice 6–12 weeks of age were treated intranasally with 50 μl of 232.4 μM synthetic 5-OP-RU in PBS, or with PBS alone, on days 0, 1, 2 and 4. On day 6, the mice were killed by cervical dislocation and the heart was perfused with 5 ml PBS prior to the removal of mediastinal lymph node. Mice were randomised and blindly allocated to groups by animal house staff prior to assignment. Once allocated, no mice were excluded for analysis.

### Cell suspensions

Thymus and spleen cell suspensions were prepared by gentle mechanical disruption of the tissues through 40 μm nylon cell strainer into ice-cold FACS buffer (PBS with 2% FBS), treated with red blood cell lysis buffer (Sigma Aldrich) then resuspended in FACS buffer. Suspensions of hepatic leukocytes were prepared by gentle mechanical disruption of the liver through 40 μm nylon cell strainer into ice-cold FACS buffer. To purify lymphocytes, cell suspensions were centrifuged in 33% Percoll (GE Healthcare) and treated with red blood cell lysis buffer before resuspending in FACS buffer.

### Flow cytometry

Cells in suspension were incubated with 7-aminoactinomycin D (7-AAD, Thermo Fisher Scientific) plus antibodies against: mouse TCRβ (H57-597, BioLegend), B220 (RA3-6B2, BioLegend, BD Biosciences), CD19 (1D3, BD Biosciences), CD4 (RM4-5, BioLegend), mouse CD1d-α-GalCer tetramer (produced in house as above), Each reagent was titrated to determine the optimal dilution. All data were acquired on an LSR Fortessa II (BD Biosciences), and analysed with FACSDiva and FlowJo (BD) software. All samples were gated on lymphocytes, with exclusion of doublets and dead cells using forward and side scatter area, forward scatter area and height and viability dye parameters, respectively.

### Immunohistology

Mouse tissues were harvested and snap frozen in Optimal Cutting Temperature (OCT, Tissue-Tek) and 6–9 μm sections cut. Before removal, mice were perfused with PBS and lungs were inflated with 33% OCT and PBS solution. Sections were treated with 100% acetone (pre-chilled to − 20 °C,) for 10 min at room temperature and air dried. Sections were blocked with 2% Bovine Serum Albumin (BSA, Sigma-Aldrich) and 10% normal mouse sera, and 10% normal goat, rat and/or donkey sera (Thermo Fisher Scientific) as required depending on the primary, secondary or tertiary antibodies used, for 1 h at room temperature. Sections were stained with various combinations of primary antibodies: anti-CD3 (500A2, eBioscience), anti-CD4 (RM4.5, eBioscience), anti-CD205 (NLDC-145, BioLegend) and anti-cytokeratin-5 (K5, EP1601Y, Abcam), or labelled antibodies: anti-TCRβ AF647 (H57-597, BioLegend), anti-CD3 AF594 or AF647 (17A2, BioLegend), anti-CD4 AF488 (RM4.5, BD Biosciences and BioLegend), anti-B220 Pacific Blue (RA3-6B2, BioLegend), anti-CD11c AF488 (N418, BioLegend), anti-CD11b biotin (M1/70, BioLegend), anti-MHC-II AF700 (M5/114.15.2, BioLegend), and CD31 AF647 (MEC13.3, BioLegend)) for 30 min at room temperature. Where required, sections were then stained with streptavidin AF680 (Thermo Fisher Scientific), donkey anti-rat AF568 (Thermo Fisher Scientific) or goat anti-hamster AF568 (Thermo Fisher Scientific) secondary polyclonal antibodies with 10% normal mouse serum and 2% BSA diluted in PBS for 30 min at room temperature. While the donkey and goat antibodies were cross absorbed to various species’ Ig, including mouse and rat by the manufacturer, the mixture of secondary antibody, normal mouse serum and BSA was made 30 min prior to use to allow for additional absorption of potential mouse Ig binding sites. When required, sections were counter stained with DAPI (Thermo Fisher Scientific). All reagents were titrated to determine the optimal dilution factor. Slides were mounted with ProLong Gold Antifade (Thermo Fisher Scientific) and left to cure overnight before imaging.

When staining with either CD1d or MR1 tetramers, the staining protocol above was modified to increase care in blocking for unwanted binding, the addition of a fixation step following tetramer staining to prevent loss of the tetramer and secondary and tertiary antibody stains to amplify tetramer signal. These modifications are as follows: After treatment with 100% acetone and air drying, 20 μg/mL of CD1d-GD3 tetramer and/or MR1-Ac-6-FP tetramer without a conjugated fluorochrome was included in the BSA and serum block to minimise non-specific CD1d- and MR1-tetramer staining. This was followed with a step to block endogenous biotin using a biotin blocking solution (Thermo Fisher Scientific), as per manufacturer’s instructions. Sections were then stained with 16 μg/mL PE- or FITC-labelled CD1d tetramers loaded with α-GalCer, GD3 or unloaded and/or PE-labelled MR1 tetramers loaded with 5-OP-RU or Ac-6-FP, for 30 min 4 °C. Sections were then fixed with 1% paraformaldehyde (Electron Microscopy Sciences) for 30 min at room temperature followed by treatment with 50 μM NH_4_Cl (Sigma-Aldrich) for 10 min at room temperature. Sections were then stained with a solution containing secondary polyclonal rabbit or goat anti-PE and/or polyclonal rabbit anti-FITC antibodies (Thermo Fisher Scientific), to bind the fluorochrome within the tetramer, and 10% normal mouse serum for 30 min at room temperature. This was followed by a tertiary stain for 30 min at room temperature involving goat anti-rabbit-AF555, donkey anti-goat-AF555 and/or donkey anti-rabbit-AF488 (Thermo Fisher Scientific) depending on the host species of the secondary antibodies used. The fluorochrome on the tertiary stain was selected to match as close as possible the emission of the fluorochrome labelling the tetramer. These secondary and tertiary antibody stains were included with the cocktail of antibodies used to stain other lineage markers and followed with a nuclear stain (DAPI) as the experiment required. Sections were mounted with ProLong Gold Antifade overnight before imaging.

Z-stack, 2 × 2 or 3 × 3 tiled images with 76.9 nm lateral and 400 nm axial voxel size and 1024 × 1024 voxel density were recorded on either LMS-700, LSM-710 or LSM-780 laser scanning inverted confocal microscopes with a 20x/0.8 NA objective (Zeiss). Fluorochromes were excited with 405, 488, 561 and 633 nm lasers. The tiles of each image were stitched together after acquisition.

### Bioimage analysis and histo-cytometry

Spectral unmixing was performed with Zen software. Image deconvoluted was performed with Huygens Professional (Scientific Volume Imaging) and images analysed with FIJI/ImageJ and Imaris (Oxford Instruments) software. Compensation post deconvolution was performed with the Spectral Unmixing FIJI/Image J plugin^68^. Colocalization analysis and calculation of Pearson correlation coefficients were performed with the Imaris Colocalization module with intensity thresholds set for each analysed channel based on negative controls for each stain. Max intensity was performed to flatten the z-stacks and all thresholding was performed in FIJI/ImageJ. Where image intensity changes were performed to reduce background autofluorescence and increase visual clarity, this was equally preformed across all images from test and control groups within an experiment.

To count the number of NKT cells within 100 μm of thymic CMJ, a FIJI/Image J script (see supplementary methods) was used. The location of NKT cells were manually determined and the region of interest (ROI) outlining the CMJ was manually drawn at the CD205 and K5 interface for each image. For analysis of thymic medullary voids, the medullary ROI were determined for each image based on the K5 and DAPI stain. A FIJI/Image J script (see supplementary methods) was used to determine, outline, and measure the thymic medullary voids. These were defined in the script as continuous areas within the thymic medulla ROI > 5000 μm^2^ lacking in K5 keratin stain.

Histocytometry was performed in a similar way to that described previously^45^. Briefly, once spectral unmixing, deconvolution and compensation were complete, cell segmentation was performed based on the DAPI stain with the Imaris surface rendering module. The mean channel intensity values, area, volume and x, y, z position data from each segmented surface were exported into FlowJo for cytometry analysis. The segmented surfaces were separated into the various cell types in Flowjo, which were plotted back to the original cell surfaces in Imaris and used to mask on specific channels to visually represent the cell types (B220 for B cells, CD3 for T cells, CD1d-α-GalCer tetramer for NKT cells, CD11c for DCs, and CD11b for Macrophages, while DAPI was used for miscellaneous cell populations).

### Statistics

Prism software (Graphpad) was used for t test, Wilcoxon, Friedman and ANOVA statistical analysis, comparison tests and the generation of *p* values.

## Supplementary Information

Supplementary Figures.

Supplementary Information.

## Data Availability

All datasets generated and/or analysed during this study are included in this published article and the supplementary information.
