# Supplementary material for: Differential location of NKT and MAIT cells within lymphoid tissue
Source: Sci Rep. 2022 Mar 8;12:4034. doi: 10.1038/s41598-022-07704-4 (PMC8904549; doi:10.1038/s41598-022-07704-4)

Supplementary Fig S1

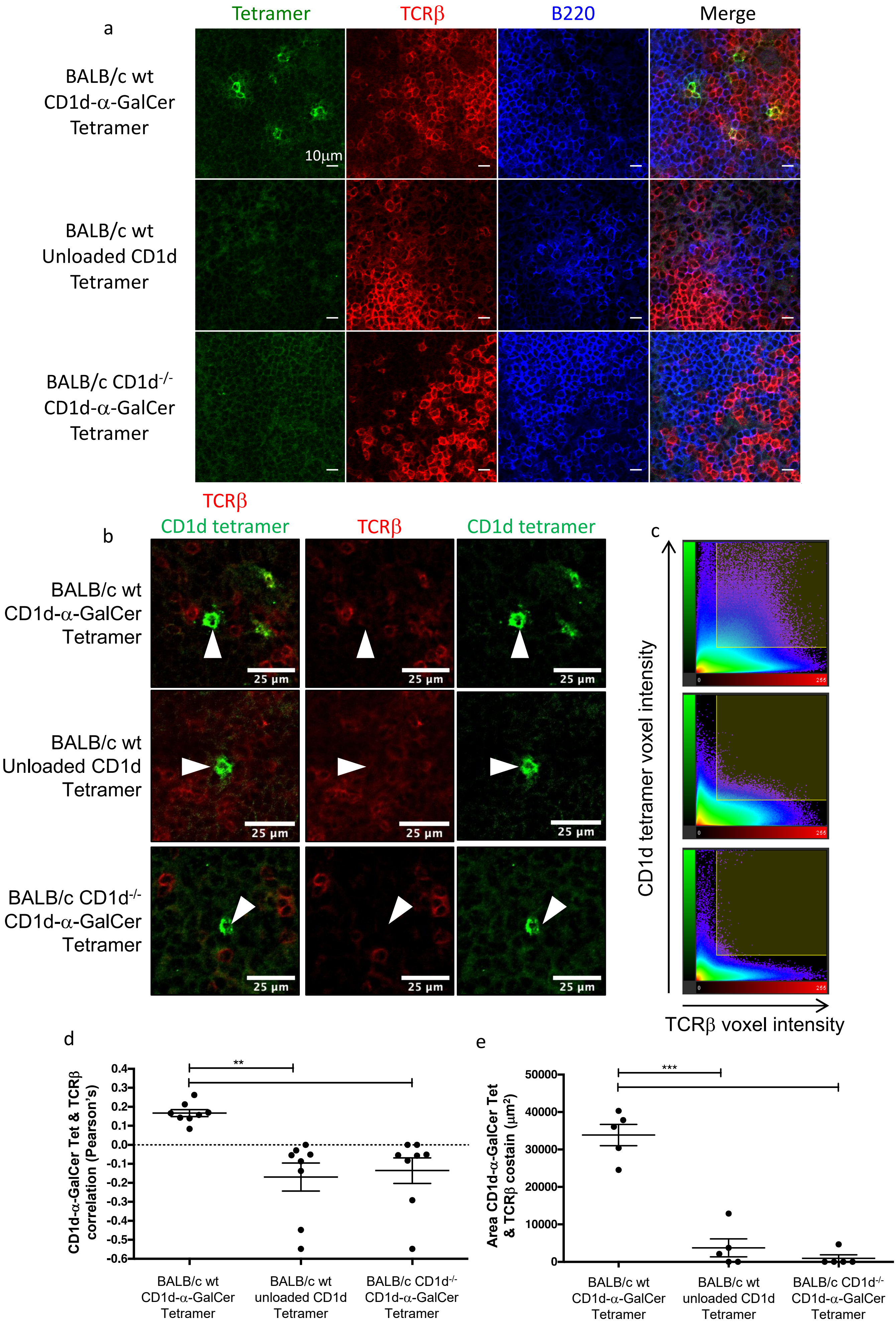

Supplementary Fig S2

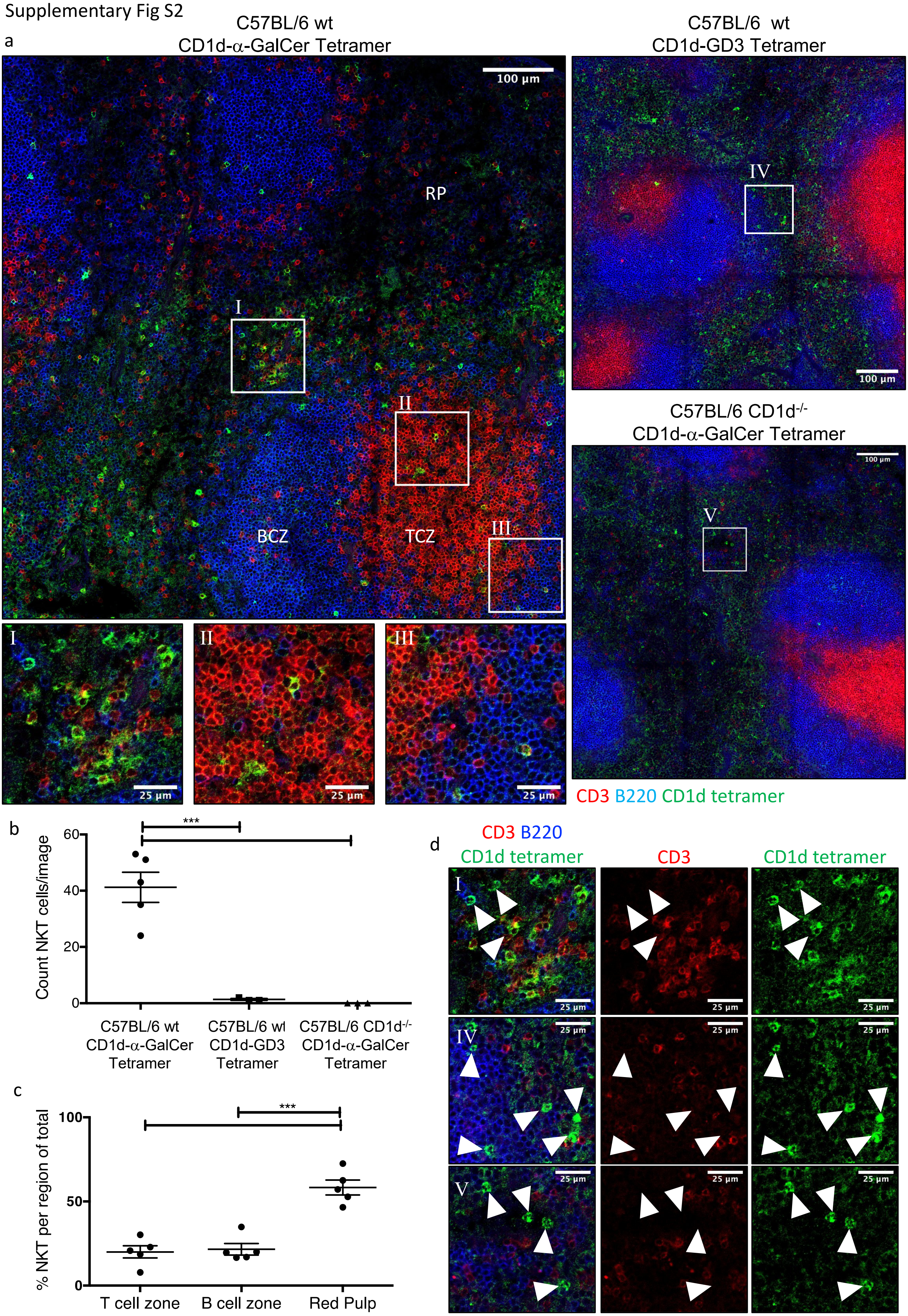

Supplementary Fig S3

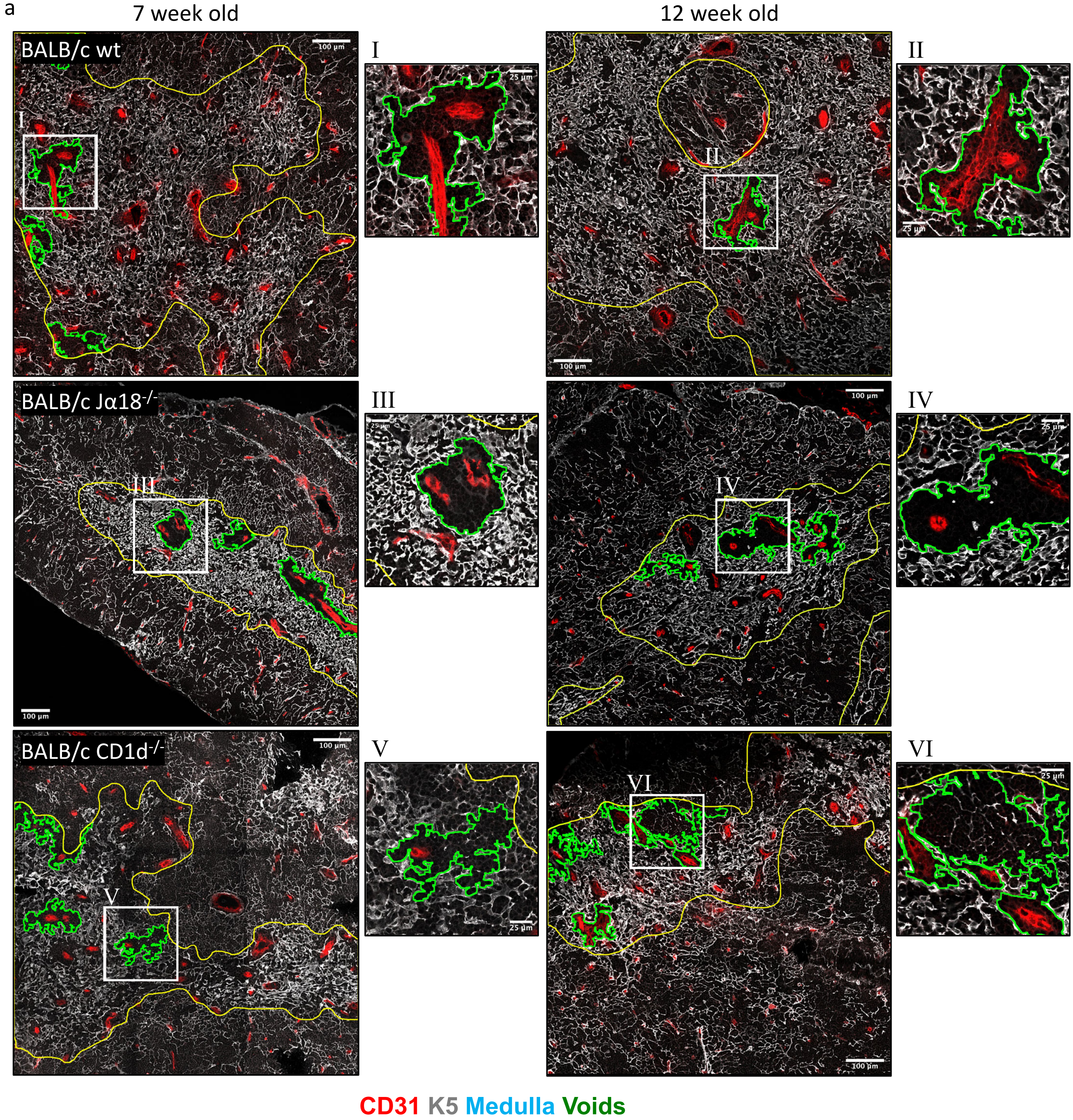

Supplementary Fig S3 continued

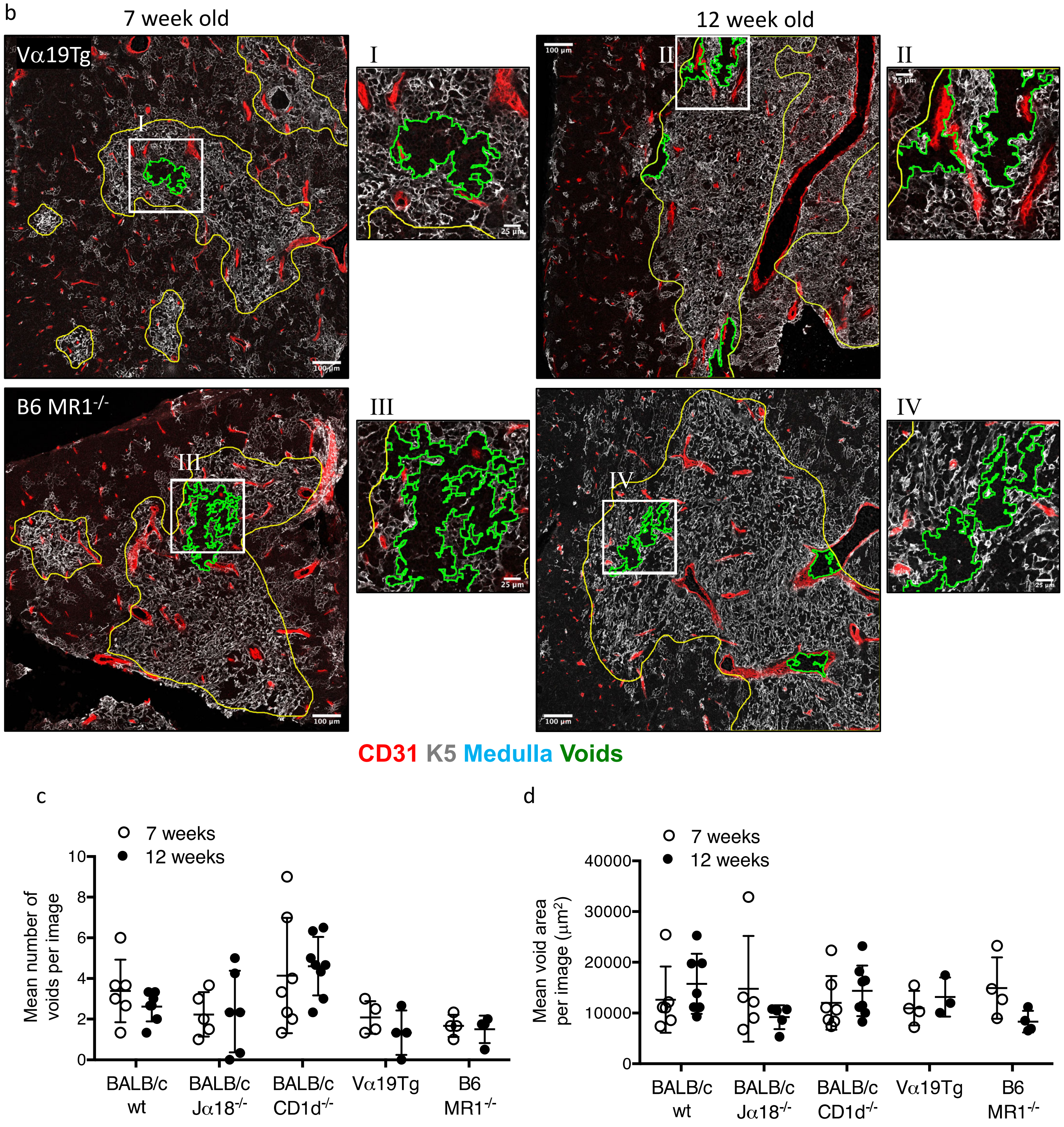

Supplementary Fig S4

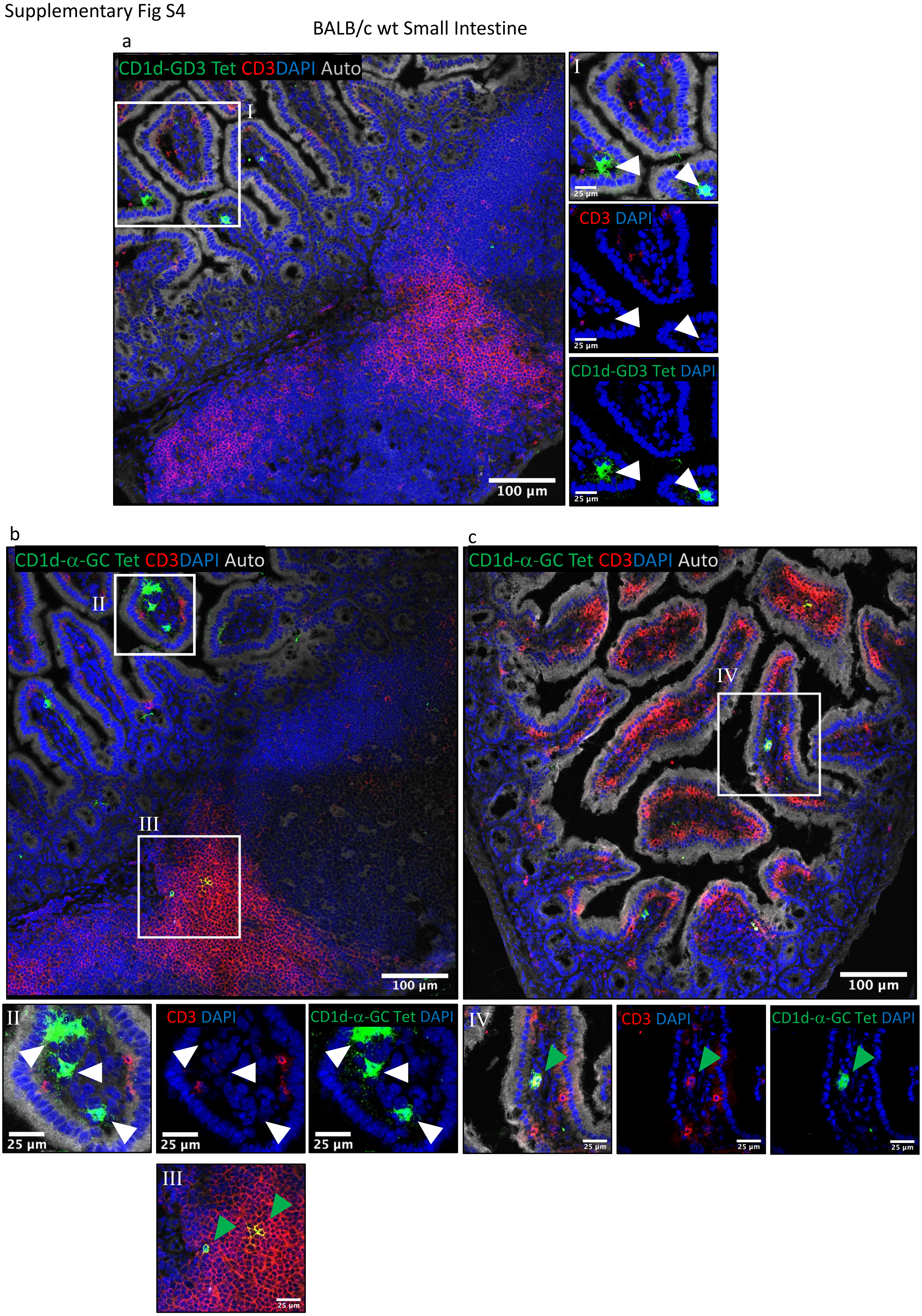

Supplementary Fig S5

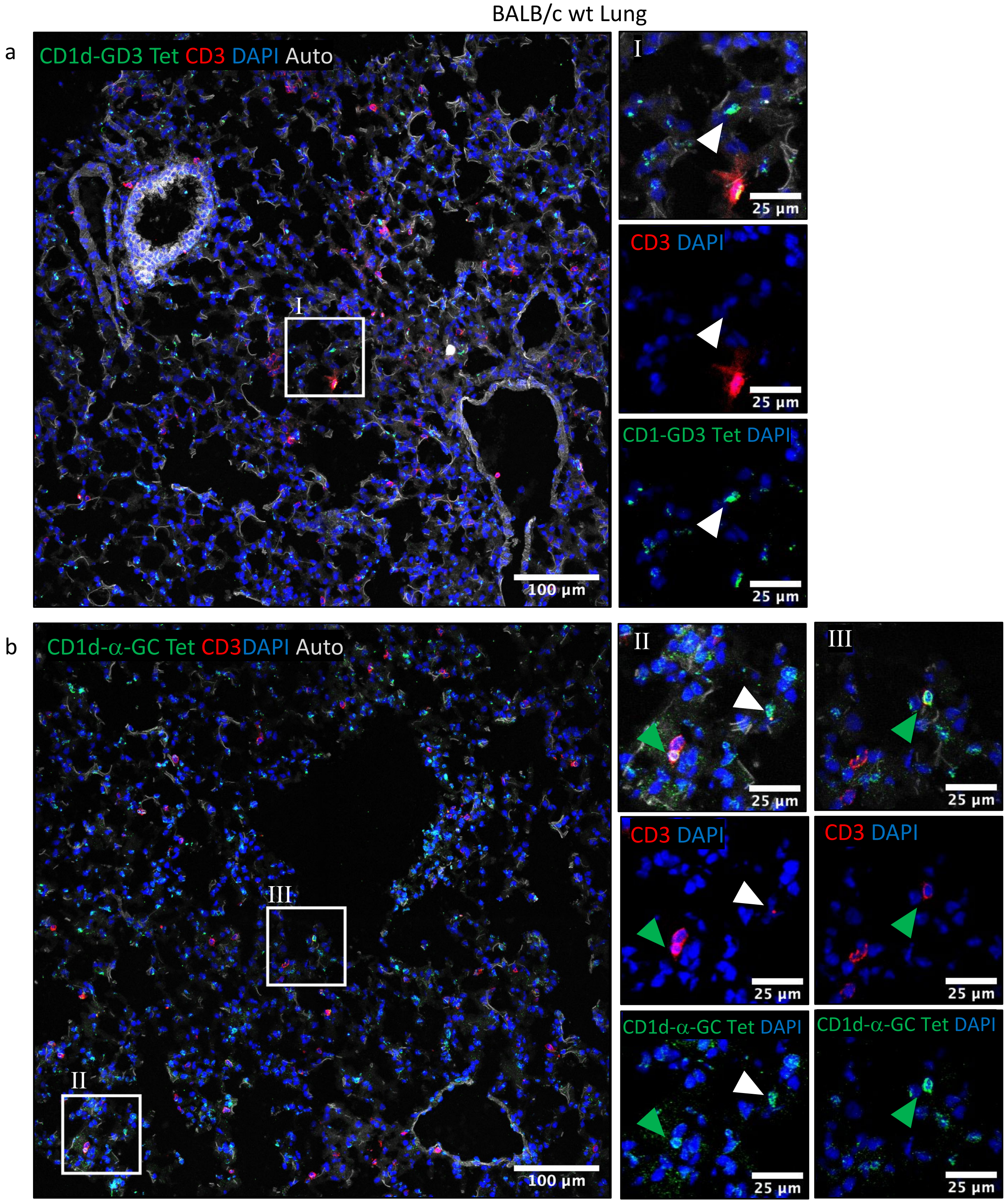

Supplementary Fig S6

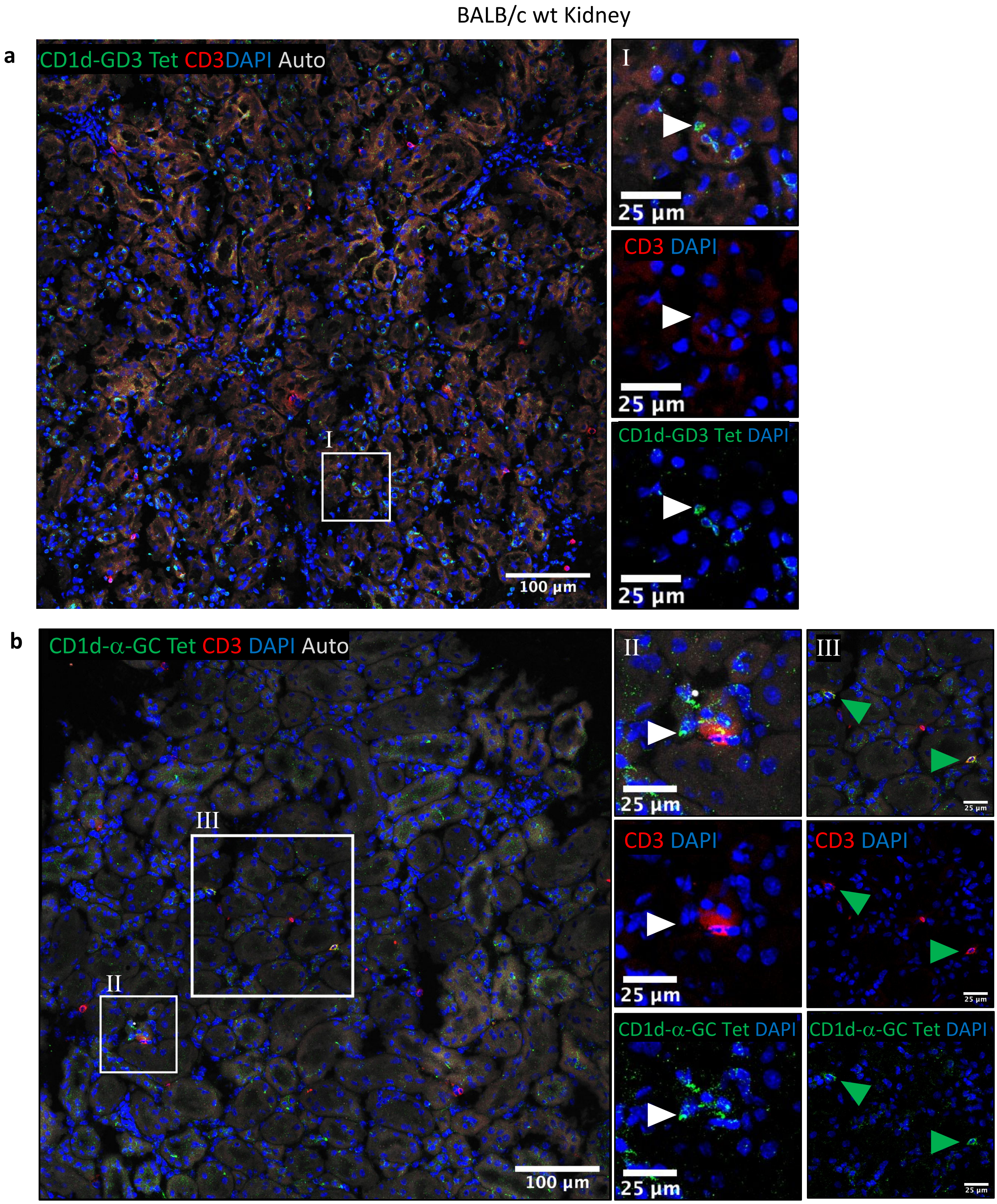

Supplementary Fig S7

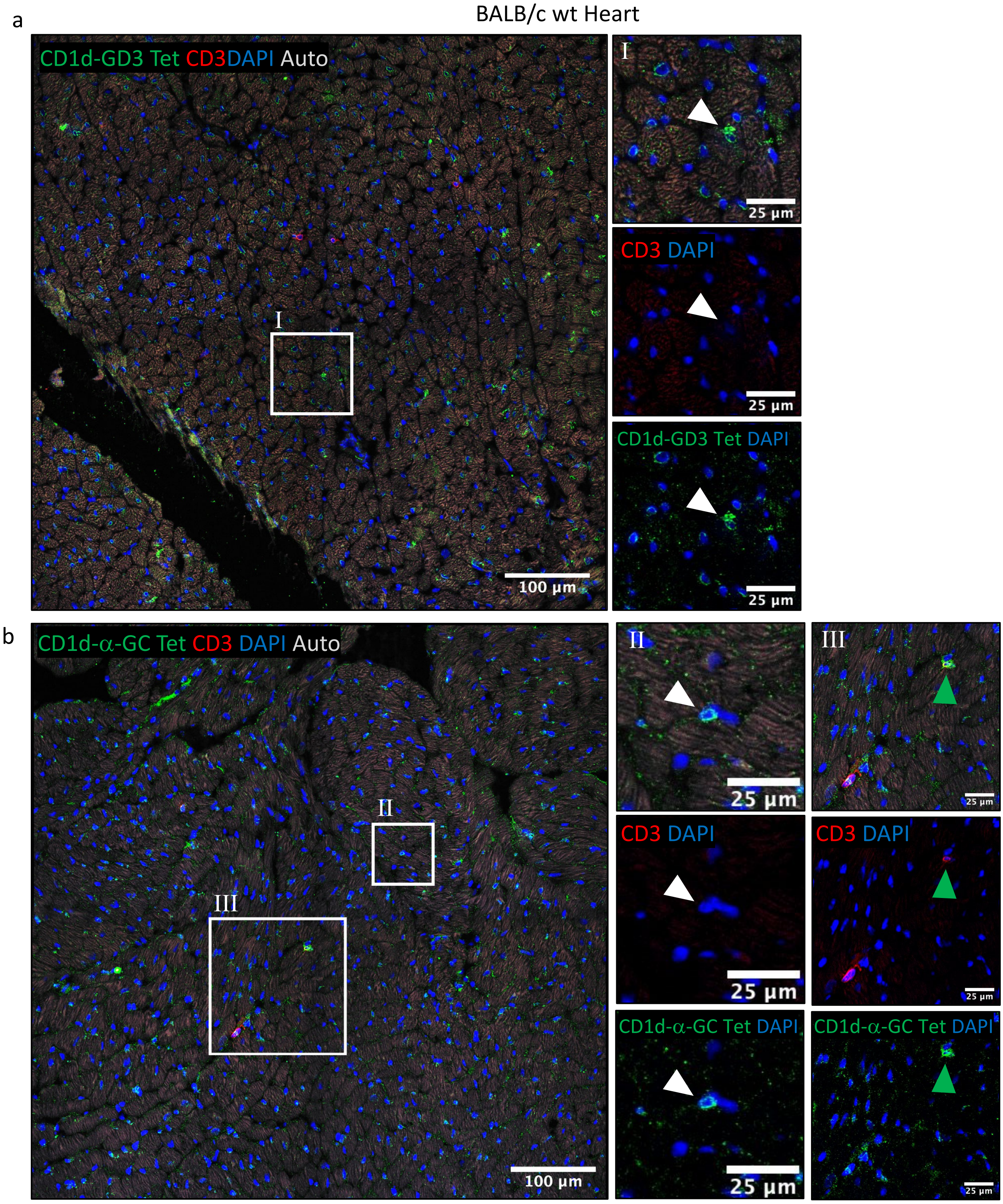

Supplementary Fig S8

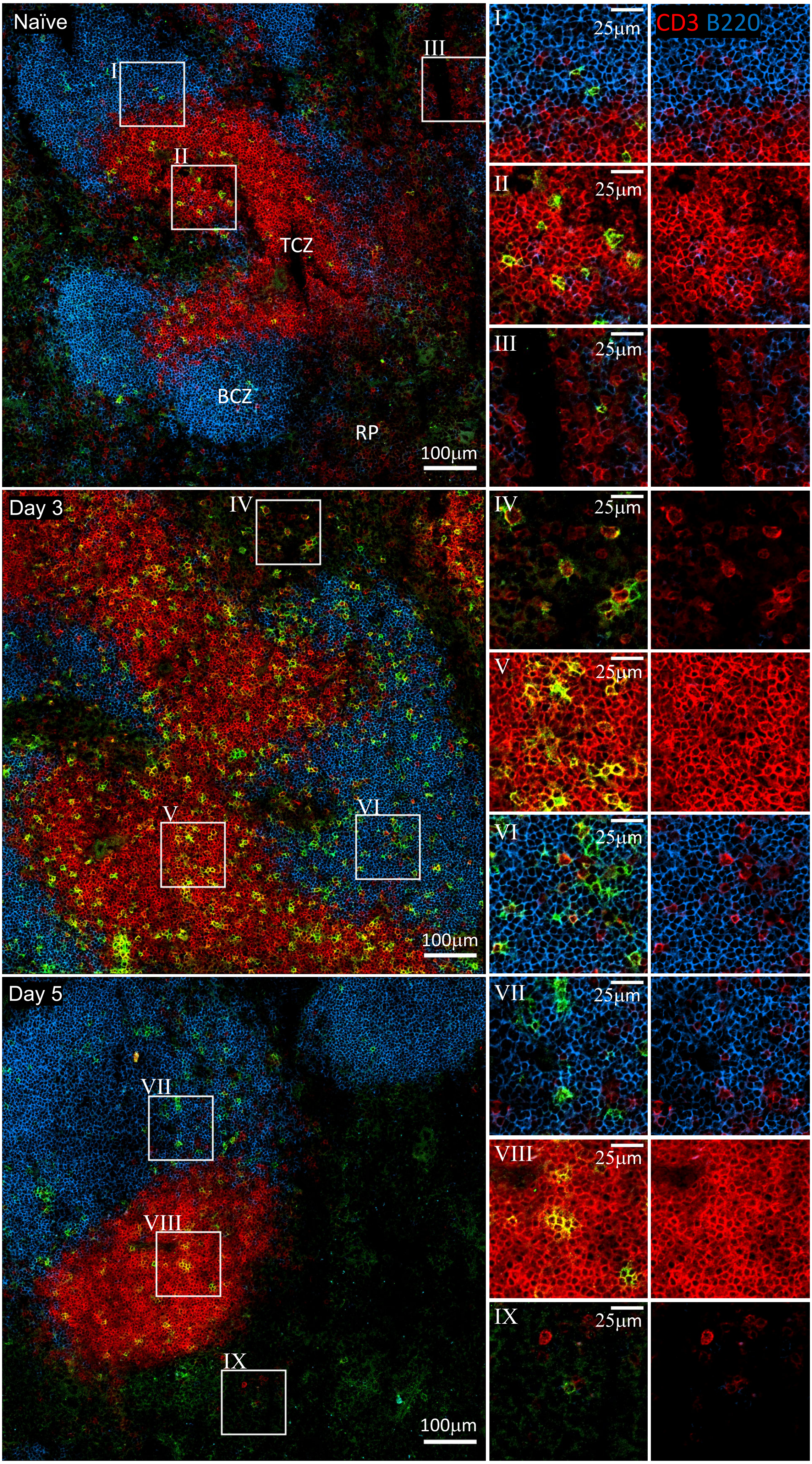

CD3 B220
CD1d-α-GalCer
Tetramer

Supplementary Fig S9

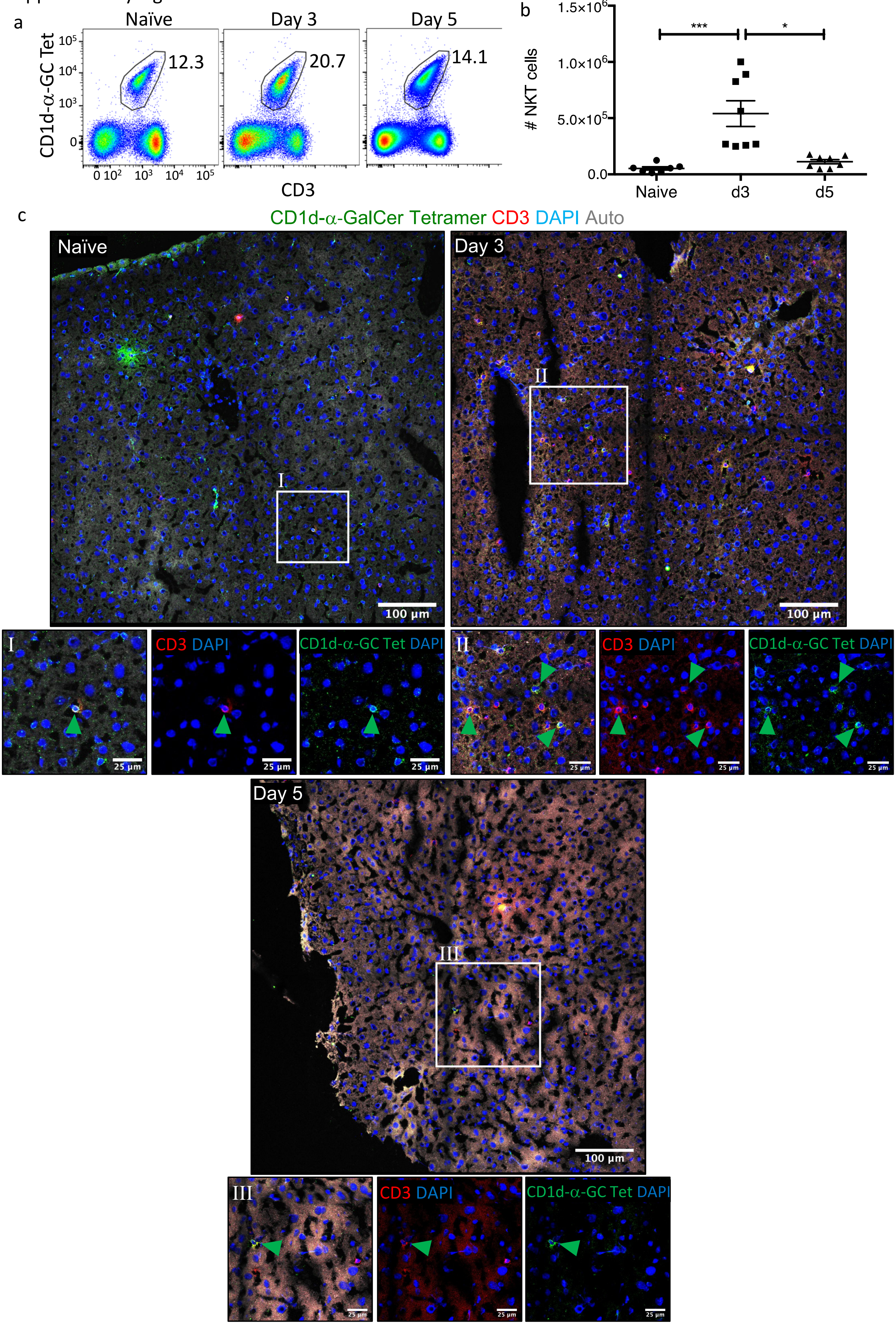

Supplementary Fig S9 continued

d

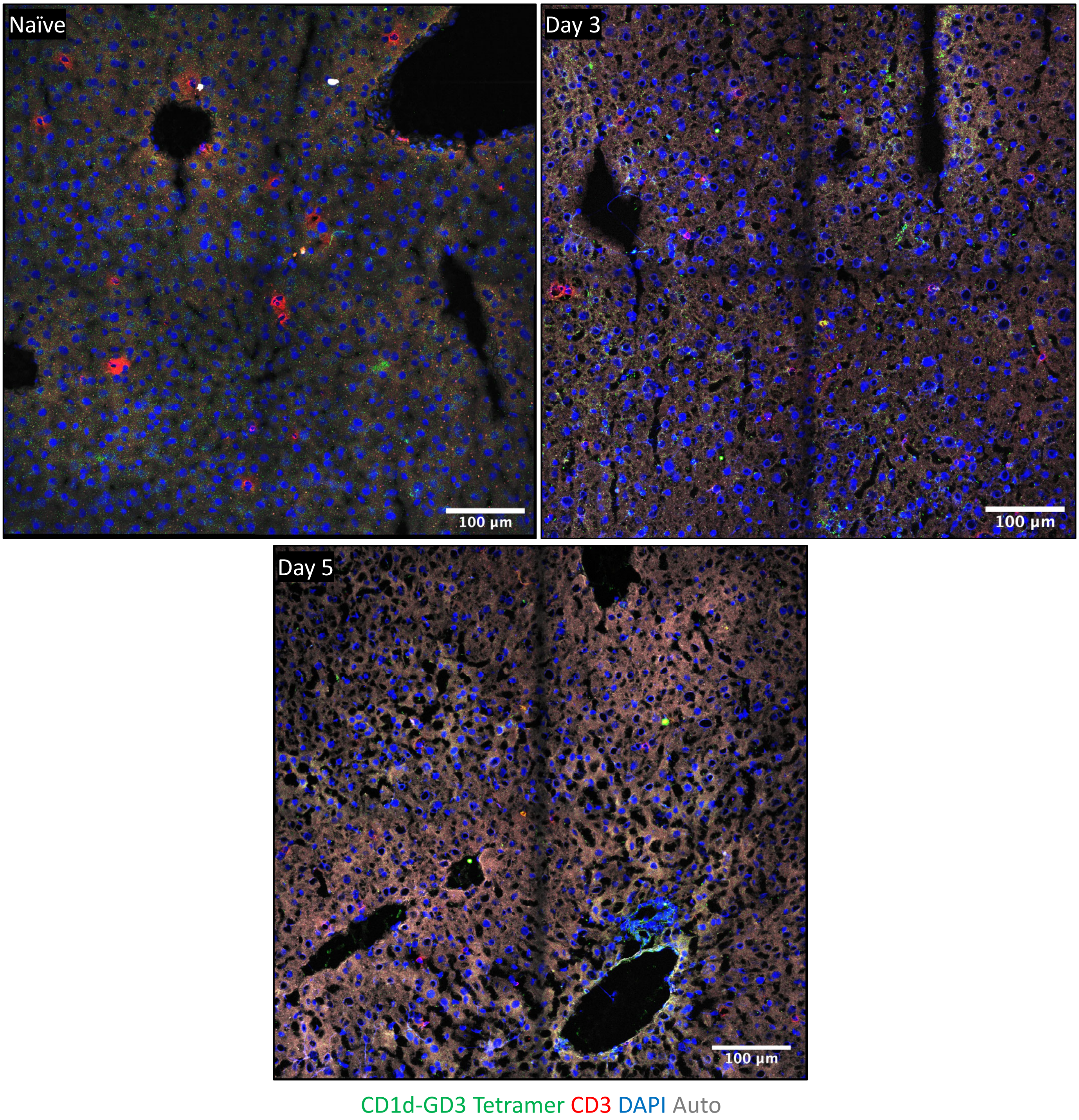

Supplementary Fig S10

a

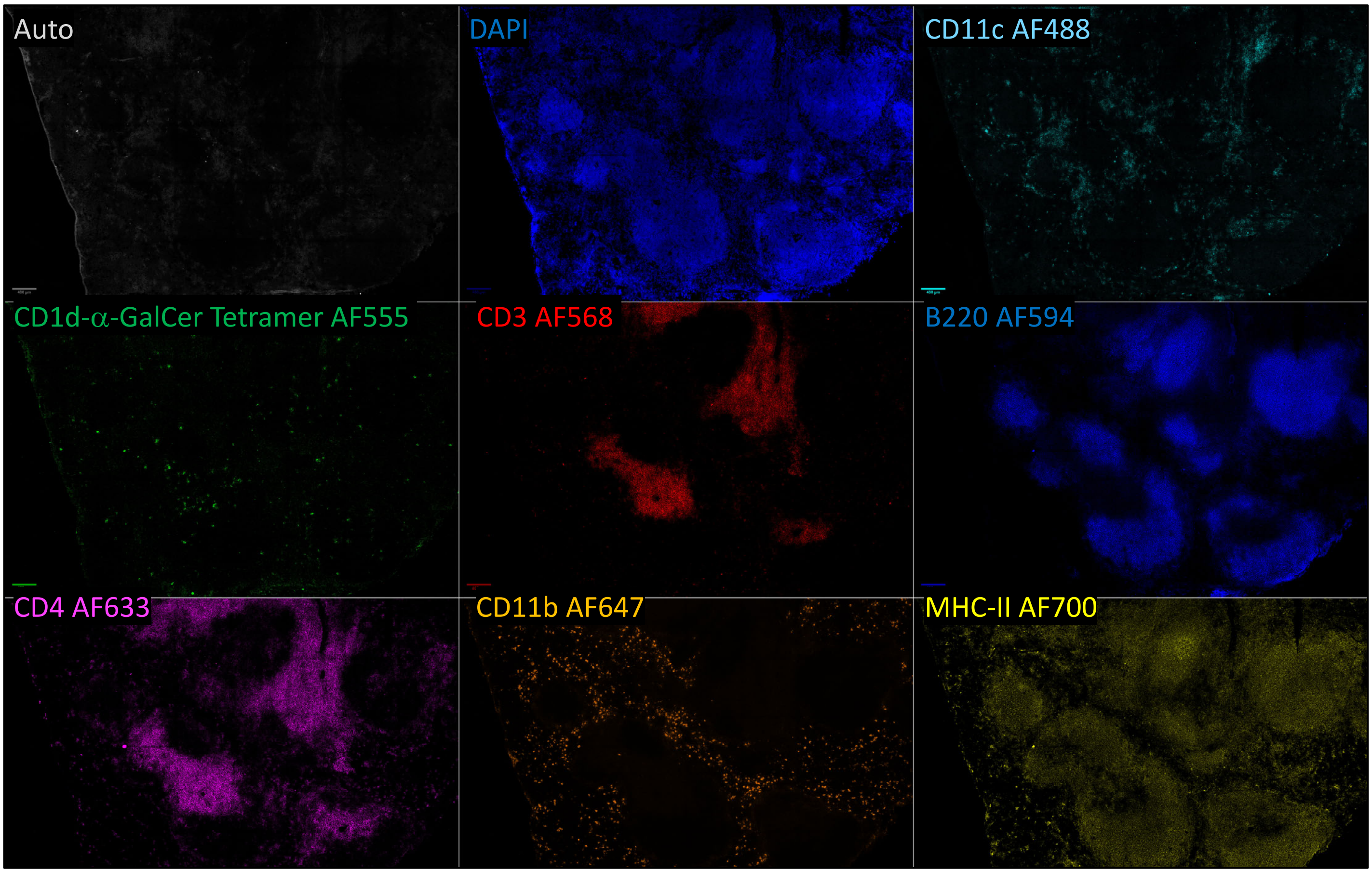

b

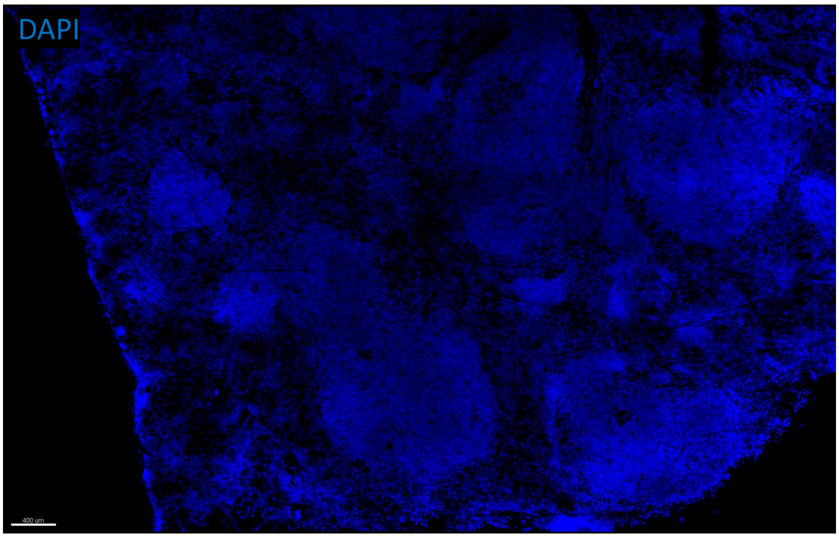

c

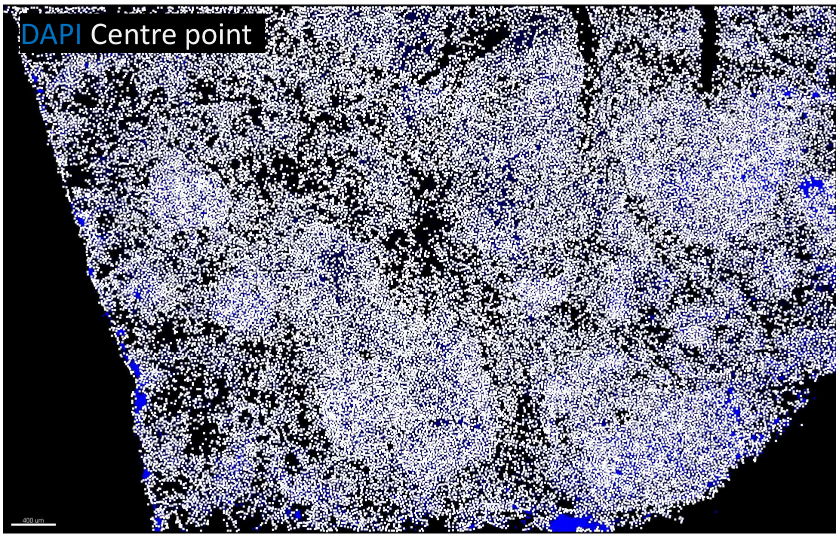

Supplementary Fig S10 continued

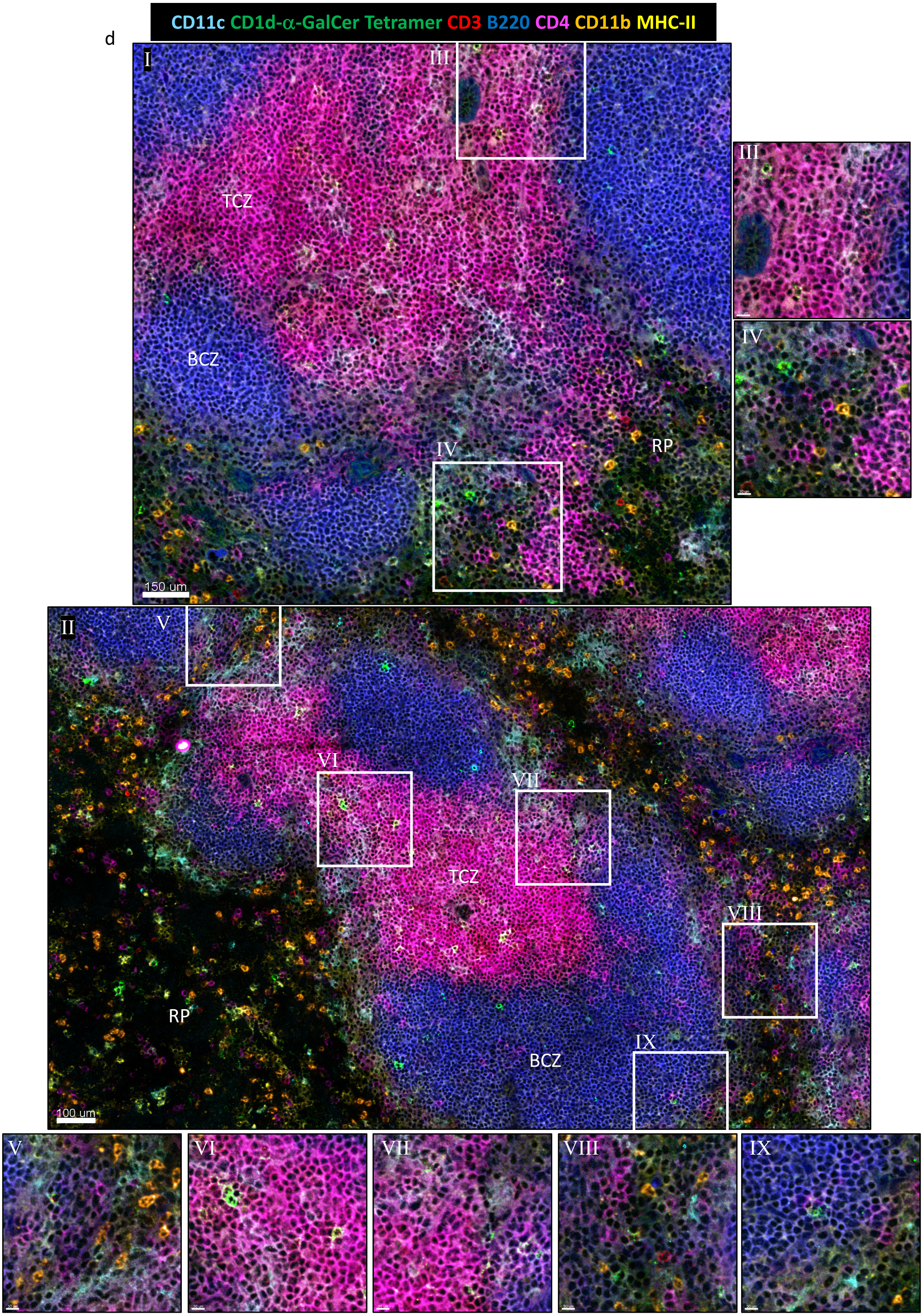

Supplementary Fig S11

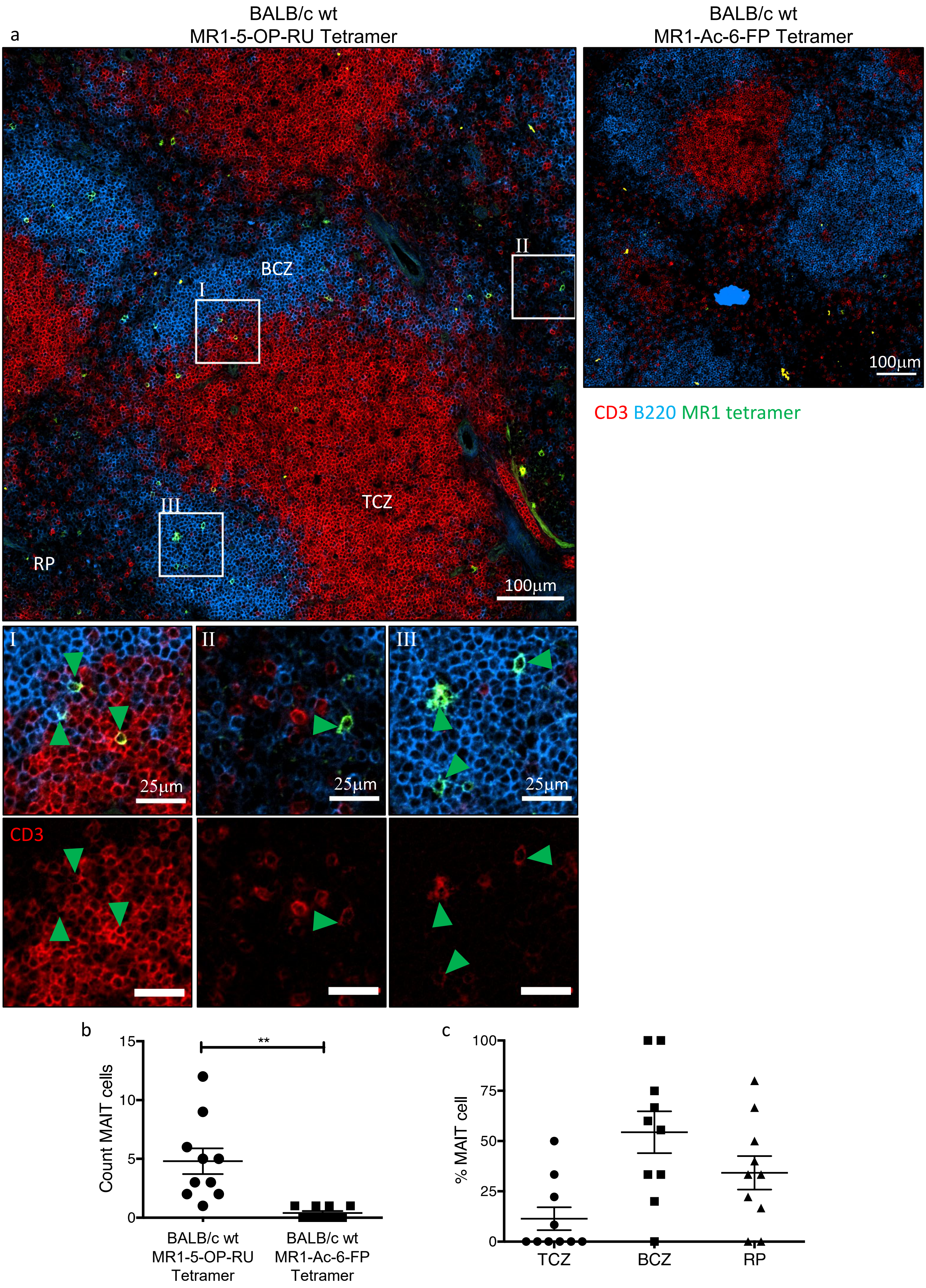

Supplementary Fig S11 continued

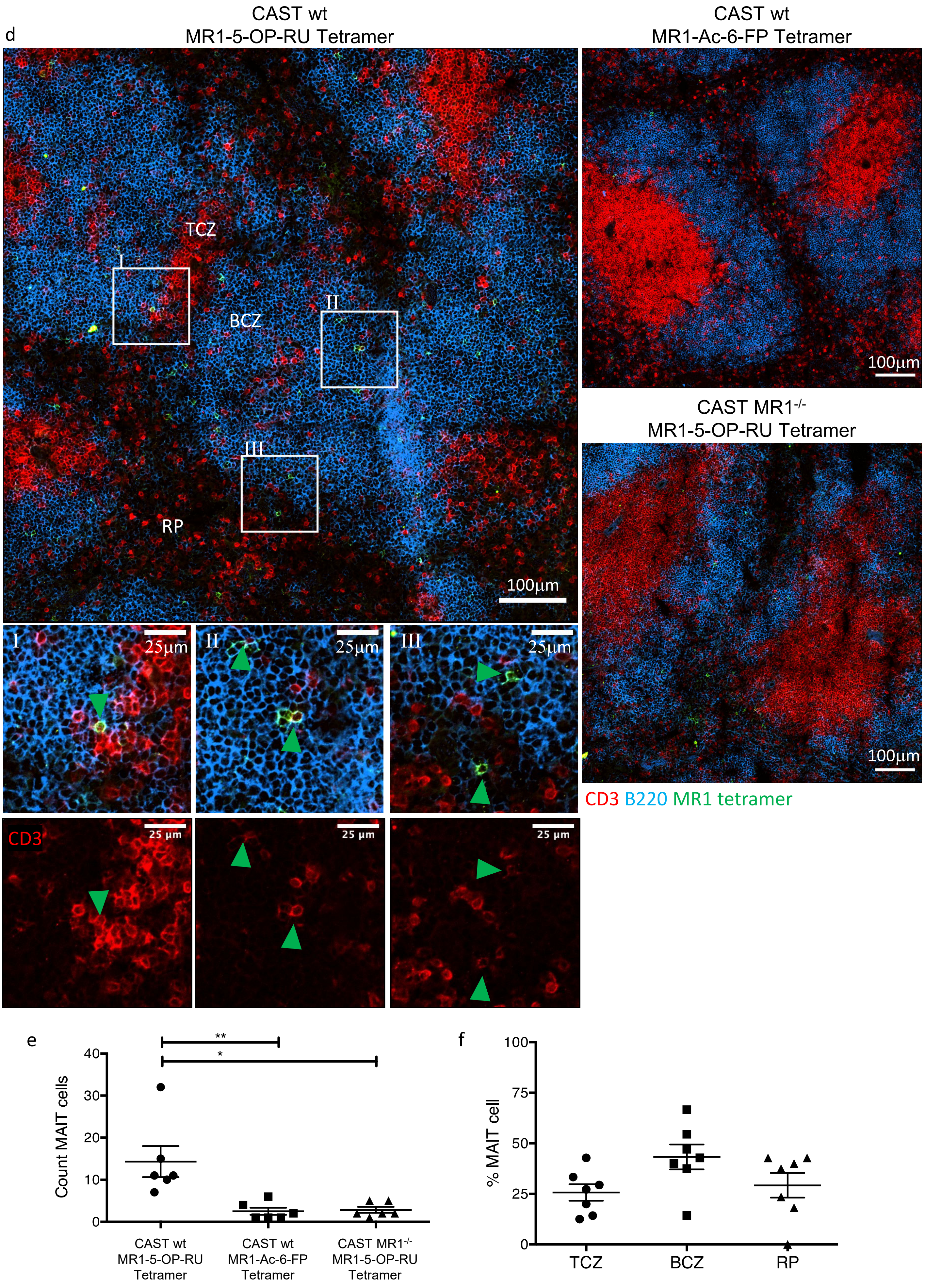

Supplementary Fig S12

a

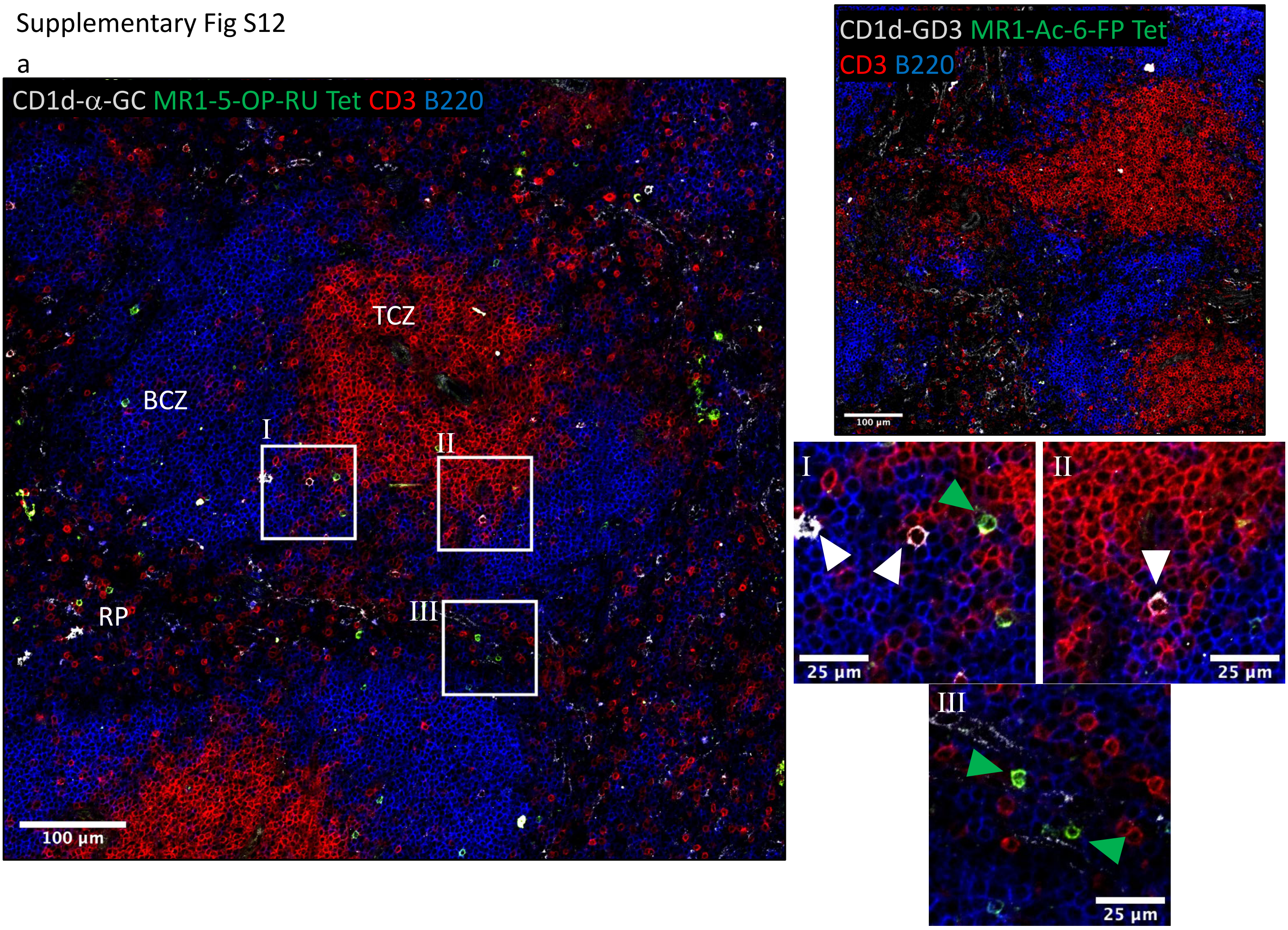

b

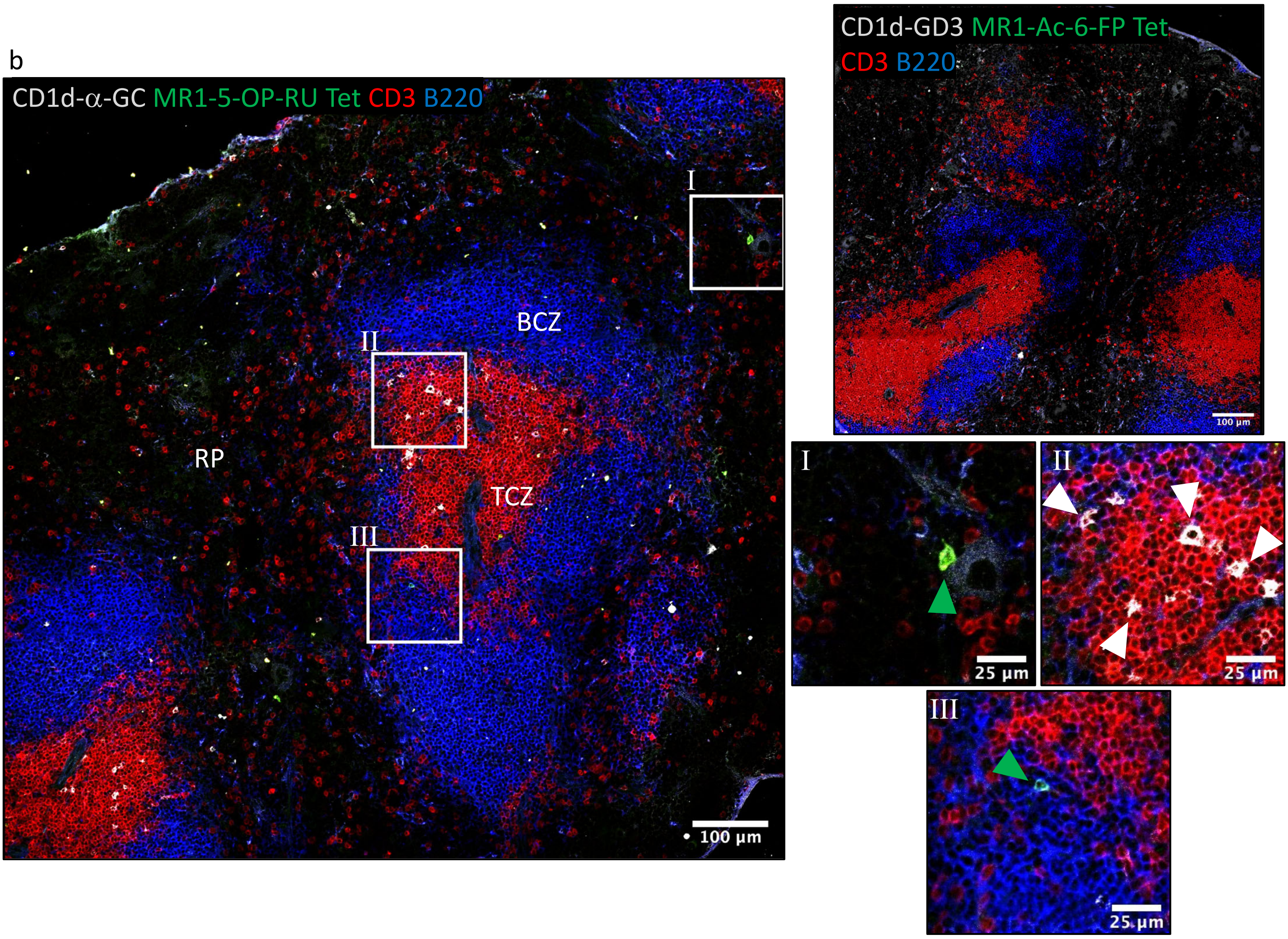

Supplementary Fig S13

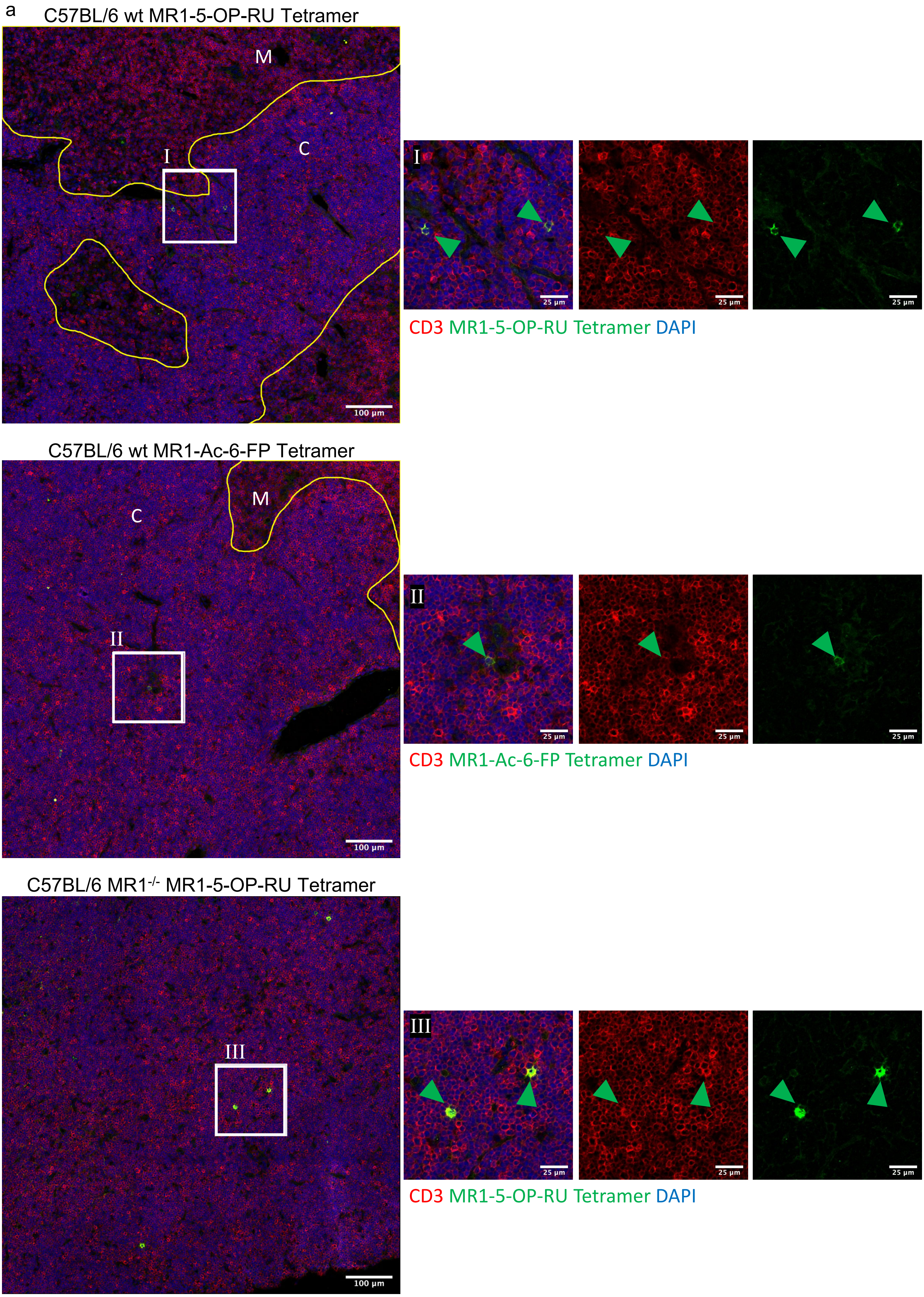

Supplementary Fig S13 Continued

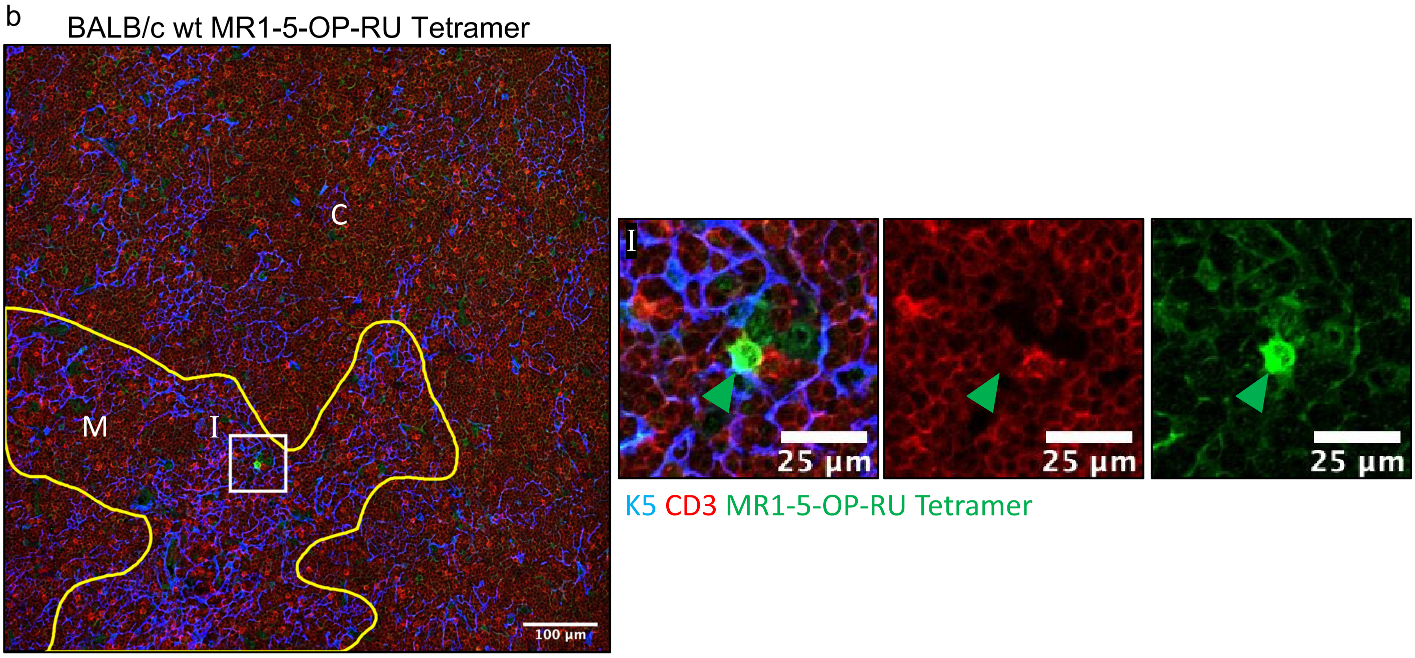

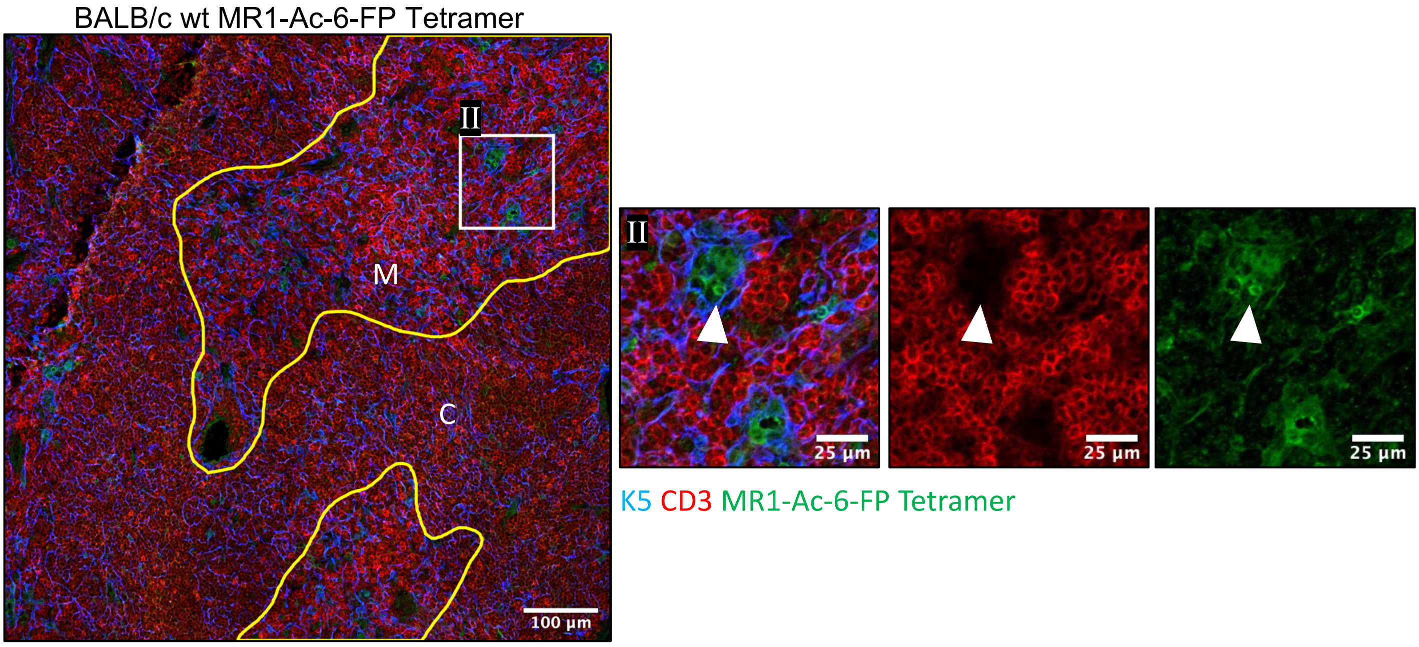

Supplementary Fig S13 continued

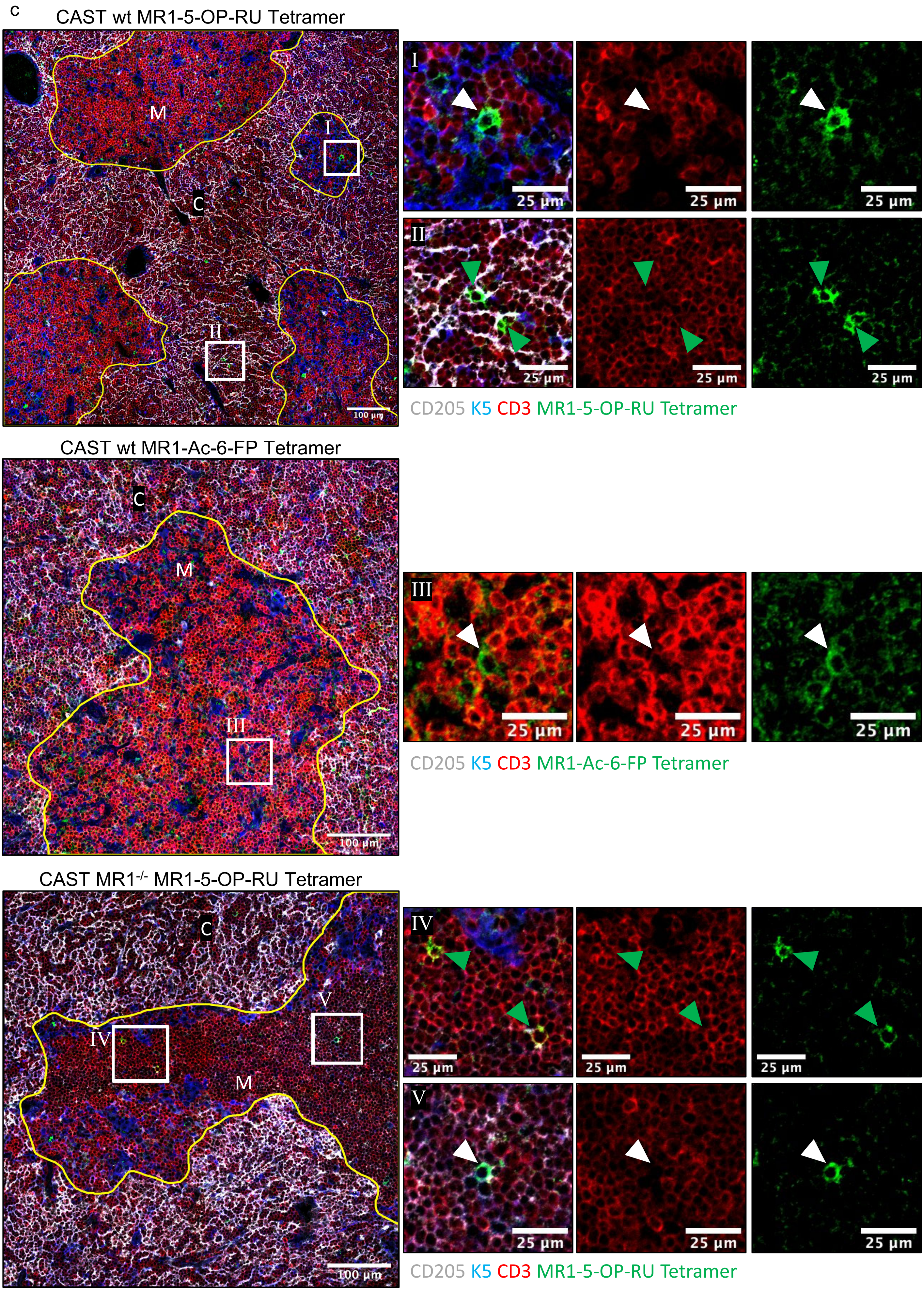

Supplementary Fig S14

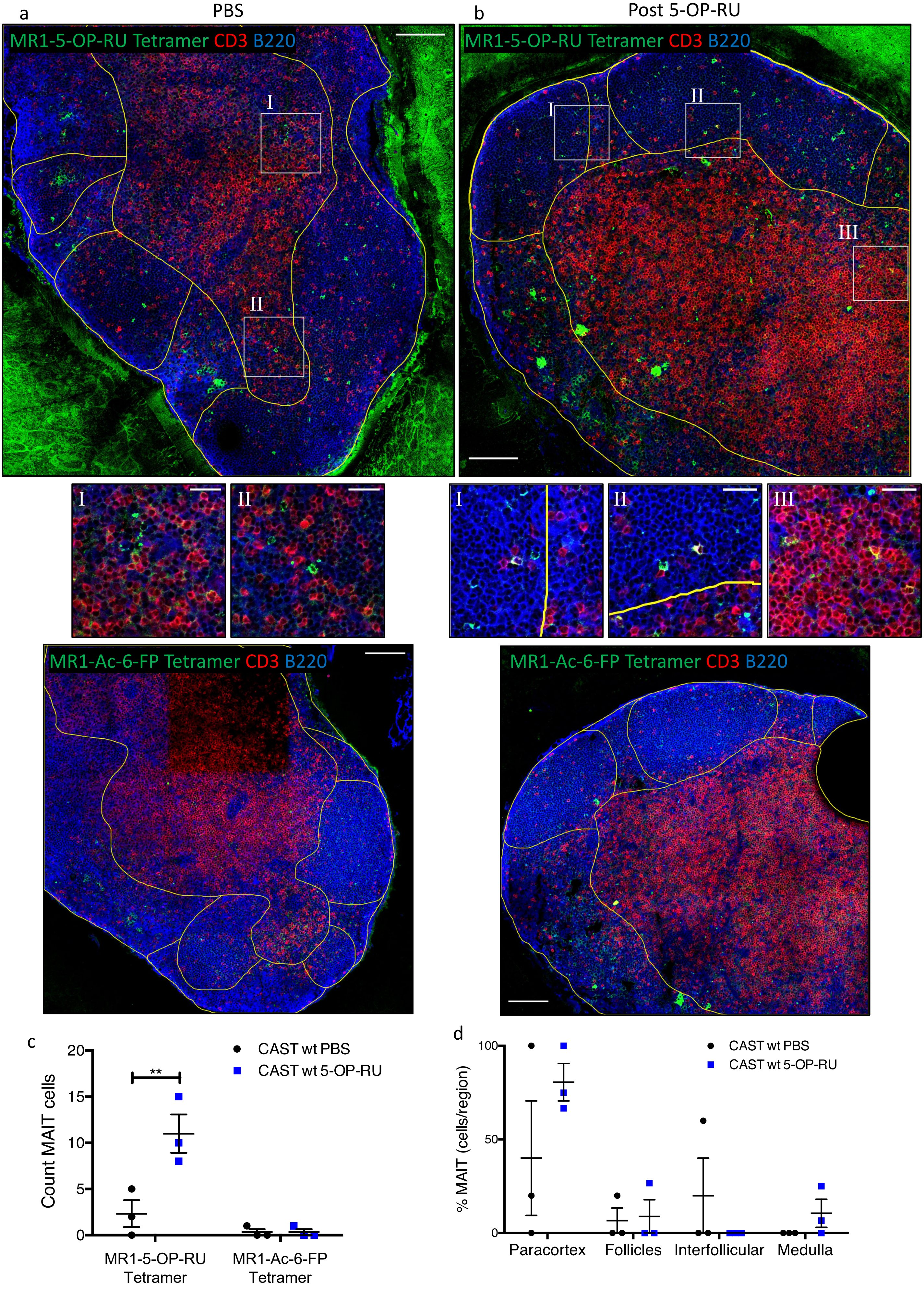

Supplement: Supplementary file 1 — Supplementary Figures. [file 41598_2022_7704_MOESM1_ESM.pdf]
