# Supplementary material for: Differential location of NKT and MAIT cells within lymphoid tissue
Source: Sci Rep. 2022 Mar 8;12:4034. doi: 10.1038/s41598-022-07704-4 (PMC8904549; doi:10.1038/s41598-022-07704-4)
Supplement: Supplementary file 2 — Supplementary Information. [file 41598_2022_7704_MOESM2_ESM.pdf]

Supplementary Information

Differential location of NKT and MAIT cells within lymphoid tissue

Darryl N. Johnson[1,2], Zheng Ruan[1], Emma V. Petley[3], Sapna Devi[1,2], Lauren E. Holz[1,2], Adam P. Uldrich[1,2], Jeffrey Y. W. Mak[4], Jyh Liang Hor[1,2], Scott N. Mueller[1,2], James McCluskey[1,2], David P. Fairlie[4], Phillip K. Darcy[3,5], Paul A. Beavis[3,5], William R. Heath[1,2], Dale I. Godfrey[1,2*]

[1]Department of Microbiology & Immunology at the Peter Doherty Institute for Infection and Immunity, The University of Melbourne, Parkville, VIC, 3010, Australia.

[2]Australian Research Council Centre of Excellence for Advanced Molecular Imaging at the University of Melbourne, VIC, 3010, Australia.

[3]Cancer Immunology Program, Peter MacCallum Cancer Centre, Melbourne, VIC, 3000, Australia,

[4]Australian Research Council Centre of Excellence in Advanced Molecular Imaging, Institute for Molecular Bioscience, The University of Queensland, Brisbane, QLD, 4072, Australia.

[5]Sir Peter MacCallum Department of Oncology, The University of Melbourne, Parkville, VIC, 3010, Australia.

* Corresponding author.

List of Contents:

Supplementary Methods

Supplementary Figure S1: *Validation of the identification of NKT cells in situ with CD1d-α-GalCer tetramer.*

Supplementary Figure S2: *Positive identification of NKT cells in situ with CD1d-α-GalCer tetramer and their location within C57BL/6 mouse spleens.*

Supplementary Figure S3: No evidence of an i*ncrease in medullary holes in thymuses of BALB/c, Vα19Tg or C57BL/6 MR1$^{-/-}$ mice.*

Supplementary Figure S4: *NKT cells within small intestine.*

Supplementary Figure S5: *NKT cells within lungs.*

Supplementary Figure S6: *NKT cells within Kidneys.*

Supplementary Figure S7: *NKT cells within Heart.*

Supplementary Fig S8: *The expansion and redistribution of NKT cells following in vivo stimulation with α-GalCer.*

Supplementary Figure S9: *CD1d-GD3 tetramer staining of BALB/c liver sections.*

Supplementary Figure S10: *Multiparameter in situ analysis of NKT cells and Antigen Presenting Cells.*

Supplementary Figure S11: *Positive identification of MAIT cells within WT BALB/c and CAST mice spleen with MR1-5-OP-RU tetramer in situ.*

Supplementary Figure S12: *CD1d and MR1 tetramer co-stain for NKT cells MAIT cells within WT C57BL/6 and BALB/c mice spleen in situ.*

Supplementary Figure S13: *No MAIT cells identified within C57BL/6, BALB/c and CAST mice thymus with MR1-5-OP-RU tetramer.*

Supplementary Figure S14: T*he expansion of MAIT cells following intranasal administration of 5-OP-RU.*

Supplementary Methods

Count cells within thymus

```
// Marco to count cells within set distance bands from the ROI. The ROI is drawn by hand based on K5 and DAPI stain to show the CMJ. A distance map is used to determine distance from the ROI. The results output uses the pre-set positions of cells and determine the area integrated of each point. Therefore, dividing the area integrated by 255 gives the number of cells in that area.
// Author: Darryl Johnson, University of Melbourne, with assistance from Ellie Cho, University of Melbourne.

// A dialog opens to let you select the source directory, directory is assigned to variable 'inputdir'
	inputdir = getDirectory("Select the input directory");

// A dialog opens to let you select the destination directory, directory is assigned to variable 'outputdir'
	outputdir = getDirectory("Select the output directory");

// Create a list of files from that directory. it comes as array[6] if there are 6 files
	filelist = getFileList(inputdir);

// Sets 16bit depth for the later MAP
	run("Options...", "iterations=1 count=1 edm=16-bit do=Nothing");

	setBatchMode(true);
// Set a loop
	for (i=0; i<filelist.length; i++){
	inputfilename = inputdir + filelist[i];

	// Open tif files in the list.
	if(endsWith(inputfilename, "tif")){

		open(inputfilename);
		fileName=getTitle();
		Name = File.nameWithoutExtension;
		inputCellPointsName = inputdir + Name+"_cellROI.roi";
		inputROIname = inputdir + Name+"_ROI.roi";

			run("Duplicate...", "duplicate channels=1");
			fileNameDuplicate=getTitle();
			selectWindow(fileNameDuplicate);

	// Makes mask of cell points
```

```
            // Opens the ROI manager and required cell points ROI.
            run("ROI Manager...");
            roiManager("reset");
            roiManager("Open", inputCellPointsName);
            roiManager("Select", 0);

      run("Create Mask");
      rename("Mask of cells");
      run("Set Measurements...", "area integrated redirect=[Mask of cells] decimal=3");

// Select the duplicated window
      selectWindow(fileNameDuplicate);
      run("Select None");

// Makes the distance map inside the ROI (edge to centre)
      // Opens the ROI manager and required medulla ROI.
            roiManager("reset");
            roiManager("Open", inputROIname);
            roiManager("Select", 0);

      run("Create Mask");
      run("Distance Map");

// Count cells within 25micron to 150micron outer bands from centre of the ROI to the edge. The thresholds
are set to the pixel width of the image (in this case 1pixel = 0.41666micros).
      // 25micron
            setThreshold(1, 60);
            run("Create Selection");
            run("Measure");
      // 50micron
            setThreshold(1, 120);
            run("Create Selection");
            run("Measure");
      // 75micron
            setThreshold(1, 180);
            run("Create Selection");
            run("Measure");
      // 100micron
            setThreshold(1, 241);
            run("Create Selection");
            run("Measure");
      // 125micron
            setThreshold(1, 301);
            run("Create Selection");
            run("Measure");
      // 150micron
            setThreshold(1, 361);
            run("Create Selection");
            run("Measure");

// count cells within inner center of medulla. Pixels set as per pixel width above.
      // 25-1000 inner center
            setThreshold(61, 3613);
            run("Create Selection");
            run("Measure");
      // 50-1000 inner center
            setThreshold(121, 3613);
```

```
            run("Create Selection");
            run("Measure");
        // 75-1000 inner center
            setThreshold(181, 3613);
            run("Create Selection");
            run("Measure");
        // 100-1000 inner center
            setThreshold(242, 3613);
            run("Create Selection");
            run("Measure");
        // 125-1000 inner center
            setThreshold(302, 3613);
            run("Create Selection");
            run("Measure");
        // 150-1000 inner center
            setThreshold(362, 3613);
            run("Create Selection");
            run("Measure");
        saveAs("Results", outputdir+Name +"-outter and inner medulla count");
        wait(100);

// Makes the distance map outside the ROI (edge of ROI to the end of the window)
        selectWindow("Mask");
        run("Invert LUT");
        run("Distance Map");

// Count cells within cortex
        setThreshold(1, 3613);
        run("Create Selection");
        run("Measure");

        saveAs("Results", outputdir+Name +"-whole count");
        wait(100);
        close("Results");

// clean up
        run("Close All");
        run("Collect Garbage");

                    }
}

setBatchMode("exit & display");
print("Done ;)");
```

Thymus hole measurement

```
//This marco opens an image and applies a pre-drawn ROI onto the image. The data outside the image is removed and the 'holes' between the keratin5 stain is meaured.
//Author: Darryl Johnson, University of Melbourne, with assistance from Ellie Cho and Robyn Esterbauer, University of Melbourne.
//Run from Plugins > Macros

//A dialog opens to let you select the source directory, directory is assigned to variable 'inputdir'
	Dialog.create("Select imput folder");
	Dialog.show();
	inputdir = getDirectory("Select the input directory");

//A dialog opens to let you select the destination directory, directory is assigned to variable 'outputdir'
	Dialog.create("Select output folder");
	Dialog.show();
	outputdir = getDirectory("Select the output directory");

//Optimise which channel to be analysed, analyse particle and threshold settings specific to your application and result of interest
//Dialog: Get values from the user
	Dialog.create("Set the parameters");
	Dialog.addString("K5 channel : ", "C1");
	Dialog.addString("DAPI channel : ", "C3");
	Dialog.addNumber("K5 Setting lower threshold : ", 0);
	Dialog.addNumber("K5 Setting upper threshold : ", 15);
	Dialog.addNumber("Minimal Hole size : ", 5000);
	Dialog.show();
	K5Channel = Dialog.getString();
	DAPIChannel = Dialog.getString();
	LowThresholdK5Setting = Dialog.getNumber();
	HighThresholdK5Setting = Dialog.getNumber();
	minHole = Dialog.getNumber();
	AnalyzeStromalHoleSettings="size="+minHole+"-Infinity circularity=0.04-1.00 include add slice";

//create a list of files from that directory and stat a loop to measure for all files.
	filelist = getFileList(inputdir);

	for (i=0; i<filelist.length; i++){
		inputfilename = inputdir + filelist[i];
	//open tif files in the list and splits channels.
		if(endsWith(inputfilename, "tif")){
		open(inputfilename);
		fileName=getTitle();
		Name = File.nameWithoutExtension;
		inputROIname = inputdir + Name+"_ROI.roi";
		run("Split Channels");

	//make result table
		title1 = "Measurement";
		title2 = "["+title1+"]";
		if (isOpen(title1)) {selectWindow(title1); run("Close"); }
		run("Table...", "name="+title2+" width=1200 height=600");
		print(title2, "\\Headings:Image Name \t Area");

			K5_stain = K5Channel + "-" + fileName;
			selectWindow(K5_stain);
```

```
			run("Duplicate...", "title=proof");run("Grays"); run("RGB Color");
			wait(100);

			DAPI_stain = DAPIChannel + "-" + fileName;
			selectWindow(DAPI_stain);
			run("Duplicate...", "title=DAPI_stain");run("Grays"); run("RGB Color");
			wait(100);

//Selects the channel window and processes for later area measurement of the holes.
//Threshold settings are set by low and high Threshold K5 Settings above.
		selectWindow(K5_stain);
		run("Threshold...");
		setThreshold(LowThresholdK5Setting, HighThresholdK5Setting);
		run("Convert to Mask");
		run("Invert LUT");
		run("Analyze Particles...", "size=10-Infinity show=Masks");

//Renames the mask that was made.
		rename("mask");
		selectWindow("mask");

//Fills gaps in the edges of the holes
		run("Options...", "iterations=6 count=1 black do=Open");

//Opens the ROI manager and required medulla ROI.
		roiManager("reset");
		roiManager("Open", inputROIname);

//make proof image for Area selection on the K5 and DAPI stains
		setForegroundColor(0,255,0);run("Line Width...", "line=5");
		selectWindow("proof");roiManager("select",0);run("Draw", "slice");
		selectWindow("DAPI_stain");roiManager("select",0);run("Draw", "slice");
		saveAs("jpg", outputdir+Name +"-DAPI.jpg"); run("Close");
		wait(100);

//Adds area of the ROI into the first line of the table
		selectWindow("mask");roiManager("Select", 0);
		run("Clear Results"); List.setMeasurements; area = List.getValue("Area"); print(title2, Name
		+ "\t" +  area );
		setBackgroundColor(0, 0, 0);

//Run MeasureHoles function
		MeasureHoles();

//Saves the results table
		selectWindow("Measurement");
		saveAs("Text", outputdir+Name +"-2.csv");
		selectWindow("Measurement");
		run("Close");

//Make proof image for ROI detected
		selectWindow("proof");
		ROI = roiManager("count"); setForegroundColor(255,0,0);run("Line Width...", "line=5");
		for(r=0;r<ROI;r++){
			roiManager("select",r);run("Draw", "slice");
			wait(100);
		}
```

```
                saveAs("jpg", outputdir+Name +"-proof.jpg"); run("Close");
        }

run("Close All");
run("Collect Garbage");
}

setBatchMode("exit & display");
print("Done");
//*----------------------------------------------------------------------------------------------------------------------------*//
//A function that measures the area of the base ROI, clears everything outside the ROI, inverts LUT, sets threshold and measures area of particles.
//Analyise particle settings are set by "AnalyzeStromaHoleSettings" veribles above.
function MeasureHoles(){
        run("Clear Outside");
        roiManager("Deselect");
        roiManager("reset");
        run("Analyze Particles...", AnalyzeStromalHoleSettings);

//and you can get your measurement to be printed out on this general table. One table is made per image
        ROI = roiManager("count");
        if(ROI>0) {
                for(r=0;r<ROI;r++){
                roiManager("select", r); run("Clear Results"); List.setMeasurements; area = List.getValue("Area");
                print(title2, Name + "\t" +  area );
                        }
                        //to save the hole ROIs
                                roiManager("deselect");
                                roiManager("Save", outputdir + Name+ "-RoiSet.zip");
                }
                else { print(title2, Name + "\t" +  "NA" );
                }
        }
```

Supplementary Fig S1: *Validation of the identification of NKT cells in situ with CD1d-α-GalCer tetramer.*

Sections from BALB/c WT or CD1d$^{-/-}$ mice spleens were stained with CD1d tetramers loaded with α-GalCer or left unloaded. **a**, Representative images showing CD1d tetramer (green), B220 (blue) and TCRβ (red) staining, scale bar 25 μm. **b** Representative image showing non-NKT (CD1d-tetramer^{+}, but CD3^{-}) staining; merged or single channel CD3 and CD1d tetramer staining. White arrows show the location of CD3^{-} non-specific CD1d tetramer stain. **c** Representative voxel density plots depicting correlation between CD1d tetramer and TCRβ staining. **d** Pearson correlation coefficients between the indicated groups and **e** area co-stained with CD3 and CD1d-α-GalCer tetramer of individual fields of view. Data n=8 combined of 3 independent experiments. Microscopy images acquired with Zen 2012 (ZEISS ZEN Microscope Software for Microscope Components) and processed with FIJI/ImageJ (Fiji (imagej.net)) and Imaris 9.3 (Microscopy Image Analysis Software - Imaris - Oxford Instruments (oxinst.com)) and figure compiled with Microsoft PowerPoint (Microsoft PowerPoint Slide Presentation Software | Microsoft 365). Mean ± SEM, Kruskal-Wallis with Dunn's post-hoc test, ** = $p < 0.01$, *** = $p < 0.005$.

Supplementary Fig S2: *Positive identification of NKT cells in situ with CD1d-α-GalCer tetramer and their location within C57BL/6 mouse spleens.*

Spleen sections from C57BL/6 WT or CD1d$^{-/-}$ mice stained with CD1d tetramers loaded with α-GalCer or GD3 and anti-B220, CD3 and CD4 antibodies. **a** Representative images showing CD1d tetramer (green), B220 (blue) and CD3 (red) staining within splenic red pulp (RP), B cell zone (BCZ) and T cell zone (TCZ). **b** Count of CD1d-α-GalCer tetramer^{+}CD3^{+} NKT cells per 0.63 mm^{2} image and **c** proportion of NKT cells within each region of the total number of NKT cells per image, mean ± SEM. **d** Zoomed regions from C57BL/6 WT and CD1d$^{-/-}$ stained with α-GalCer or GD3 loaded CD1d tetramer showing non-NKT (CD1d-tetramer^{+}, but CD3^{-}) staining; merged or single channel CD3 and CD1d tetramer staining. White arrows show the location of CD3^{-} non-specific tetramer stain. Microscopy images acquired with Zen 2012 (ZEISS ZEN Microscope Software for Microscope Components) and processed with FIJI/ImageJ (Fiji (imagej.net)) and Imaris 9.3 (Microscopy Image Analysis Software - Imaris - Oxford Instruments (oxinst.com)) and figure compiled with Microsoft PowerPoint (Microsoft PowerPoint Slide Presentation Software | Microsoft 365). Data n = 3 to 5 mice from one experiment. Mean SEM, one-way ANOVA with Tukey post-hoc test, *** = $p < 0.005$.

Supplementary Fig S3: No evidence of an i*ncrease in medullary voids in thymuses of BALB/c, Vα19Tg or C57BL/6 MR1$^{-/-}$ mice.*

Sections of (**a**) BALB/c WT, BALB/c Jα18$^{-/-}$ and BALB/c CD1d$^{-/-}$ and (**a**) Vα19Tg and C57BL/6 MR1$^{-/-}$ thymuses stained with CD31 (Red), cytokeratin 5 (K5, Gray) and DAPI (not shown). **a** and **b** Representative images showing medulla (yellow line) and medullary voids (green lines). Medulla determined from the K5 and DAPI stains and medullary voids determined with the use of an ImageJ script and defined as areas >5000 μm^{2} lacking K5 stain and within the medulla. Scale bar 100 μm of each main image and 25 μm of zoom region. **c** Mean number of voids and **d** mean void area per mouse. Microscopy

images acquired with Zen 2012 (ZEISS ZEN Microscope Software for Microscope Components) and processed with FIJI/ImageJ (Fiji (imagej.net)) and figure compiled with Microsoft PowerPoint (Microsoft PowerPoint Slide Presentation Software | Microsoft 365). Symbols represent means of 3 images per mouse thymic section, n = 3-4 mice. One experiment for each Vα19Tg and $MR1^{-/-}$ mice and 2 independent experiments for NOD mice. Mean ± SEM.

Supplementary Fig S4: *NKT cells within small intestine.*

BALB/c WT small intestine sections stained with CD3, DAPI and either (**a**) CD1d-GD3 tetramer or (**b** and **c**) CD1d-α-GalCer tetramer. Representative image showing CD1d tetramer (green), CD3 (red), DAPI (blue) and autofluorescence (Auto, gray), scale bar 100 μm, with corresponding zoomed regions of interest, scale bar 25 μm. White arrows non-NKT ($tetramer^+$, but $CD3^-$) green arrows NKT cells ($tetramer^+$, but $CD3^+$). Microscopy images acquired with Zen 2012 (ZEISS ZEN Microscope Software for Microscope Components) and processed with FIJI/ImageJ (Fiji (imagej.net)) and figure compiled with Microsoft PowerPoint (Microsoft PowerPoint Slide Presentation Software | Microsoft 365). Images representative of 2 independent experiments with 2 mice in each, Tet = Tetramer.

Supplementary Fig S5: *NKT cells within lung.*

Lung sections from BALB/c WT mice stained with CD3, DAPI and either (**a**) CD1d-GD3 tetramer or (**b**) CD1d-α-GalCer tetramer. Representative image showing CD1d tetramer (green), CD3 (red), DAPI (blue) and autofluorescence (Auto, gray), scale bar 100 μm, with corresponding zoomed regions of interest, scale bar 25 μm. White arrows non-NKT ($tetramer^+$, but $CD3^-$) green arrows NKT cells ($tetramer^+$, but $CD3^+$). Microscopy images acquired with Zen 2012 (ZEISS ZEN Microscope Software for Microscope Components) and processed with FIJI/ImageJ (Fiji (imagej.net)) and figure compiled with Microsoft PowerPoint (Microsoft PowerPoint Slide Presentation Software | Microsoft 365). Images representative of 2 independent experiments with 2 mice in each, Tet = Tetramer.

Supplementary Fig S6: *NKT cells within kidney.*

7μm sections from BALB/c WT kidneys stained with CD3, DAPI and either (**a**) CD1d-GD3 tetramer or (**b**) CD1d-α-GalCer tetramer. Representative image showing CD1d tetramer (green), CD3 (red), DAPI (blue) and autofluorescence (Auto, gray), scale bar 100 μm, with corresponding zoomed regions of interest, scale bar 25 μm. White arrows non-NKT ($tetramer^+$, but $CD3^-$) green arrows NKT cells ($tetramer^+$, but $CD3^+$). Microscopy images acquired with Zen 2012 (ZEISS ZEN Microscope Software for Microscope Components) and processed with FIJI/ImageJ (Fiji (imagej.net)) and figure compiled with Microsoft PowerPoint (Microsoft PowerPoint Slide Presentation Software | Microsoft 365). Images representative of 2 independent experiments with 2 mice in each, Tet = Tetramer.

Supplementary Fig S7: *NKT cells within heart.*

Sections from BALB/c WT heart stained with CD3, DAPI and either (**a**) CD1d-GD3 tetramer or (**b**) CD1d-α-GalCer tetramer. Representative image showing CD1d tetramer (green), CD3 (red), DAPI (blue) and

autofluorescence (Auto, gray), scale bar 100 μm, with corresponding zoomed regions of interest, scale bar 25 μm. White arrows non-NKT (tetramer$^+$, but CD3$^-$) green arrows NKT cells (tetramer$^+$, but CD3$^+$). Microscopy images acquired with Zen 2012 (ZEISS ZEN Microscope Software for Microscope Components) and processed with FIJI/ImageJ (Fiji (imagej.net)) and figure compiled with Microsoft PowerPoint (Microsoft PowerPoint Slide Presentation Software | Microsoft 365). Images representative of 2 independent experiments with 2 mice in each, Tet = Tetramer.

Supplementary Fig S8: *The expansion and redistribution of NKT cells following in vivo stimulation with α-GalCer.*

As in Fig 5c, BALB/c WT mice were injected intraperitoneally with 2 μg of α-GalCer or left unstimulated. On days 3 and 5 post injection the spleens were harvested, samples were snap frozen, cut into 7μm sections and stained with B220 and CD3 antibodies and CD1d-α-GalCer tetramer for histological analysis. Representative image showing CD1d-α-GalCer tetramer (green), B220 (blue) and CD3 (red) staining within the splenic T cells zone (TCZ), B cells zone (BCZ) and red pulp (RP), scale bar 100 μm. Corresponding zoomed regions of interest showing CD3 and B200 staining with tetramer (middle) or without tetramer (right), scale bar 25μm. Microscopy images acquired with Zen 2012 (ZEISS ZEN Microscope Software for Microscope Components) and processed with FIJI/ImageJ (Fiji (imagej.net)) and Imaris 9.3 (Microscopy Image Analysis Software - Imaris - Oxford Instruments (oxinst.com)) and figure compiled with Microsoft PowerPoint (Microsoft PowerPoint Slide Presentation Software | Microsoft 365). Images representative of n = 7-8 mice, 3 to 4 mice per group of 2 independent experiments.

Supplementary Fig S9: *CD1d-GD3 tetramer staining of BALB/c liver sections.*

BALB/c WT mice were treated with 2 μg α-GalCer i.p. or left unstimulated. On days 3 and 5 post injection the livers were harvested. Most of the liver of each mouse were prepared for FACS analysis and stained with anti-mouse B220 and CD3 antibodies and CD1d-α-GalCer tetramer. One lobe of each liver was snap frozen cut into 7μm sections and stained with CD1d-α-GalCer tetramer, anti-CD3 antibody and counterstained with DAPI. **a** Representative FACS plots and (**b**) mean number of CD3$^+$CD1d-α-GalCer tetramer$^+$ cells for each time point. Representative image showing CD3 (red), DAPI (blue) and autofluorescence (gray) and either (**c**) α-GalCer- or (**d**) GD3-loaded CD1d tetramer (green), scale bar 100 μm and zoom regions 25 μm. Microscopy images acquired with Zen 2012 (ZEISS ZEN Microscope Software for Microscope Components) and processed with FIJI/ImageJ (Fiji (imagej.net)) and figure compiled with Microsoft PowerPoint (Microsoft PowerPoint Slide Presentation Software | Microsoft 365). Symbols represent each mouse of n = 7-8 mice per group combined from 2 independent experiments, Kruskal-Wallis with Dunn's post-hoc test, * = $p < 0.05$, *** = $p < 0.005$. Tet = Tetramer.

Supplementary Fig S10: *Multiparameter in situ analysis of NKT cells and antigen presenting cells.*

Spleen sections from BALB/c WT mice were cut and stained with anti-mouse B220, CD3, CD4, CD11b, CD11c, MHC-II antibodies, CD1d-α-GalCer tetramer followed by DAPI counterstain. Following spectral

imaging, linear unmixing and deconvolution was performed followed by conventional compensation. **a** Montage of the resulting separated channels; top row Autofluorescence (Auto, gray), DAPI (blue) and CD11c (cyan); middle row CD1d-α-GalCer (green) CD3 (red) and B220 (blue); bottom row CD4 (purple), CD11b (orange) and MHC-II (yellow). Single cell masks were made based on the DAPI stain and staining data of the segmented cells exported for histo-cytometry analysis. **b** Representative image of the DAPI channel and **C** centre points resulting from cell segmentation based on the DAPI stain. **d** Corresponding images to Fig 4D showing the original stains; CD11c (cyan), CD1d-α-GalCer (green), CD3 (red), B220 (blue), CD4 (purple), CD11b (orange) and MHC-II (yellow). Scale bar of image in A, B, C 400 μm, D I and II 150 μm and III to IX 30 μm. Microscopy images acquired with Zen 2012 (ZEISS ZEN Microscope Software for Microscope Components) and processed with FIJI/ImageJ (Fiji (imagej.net)), Imaris 9.3 (Microscopy Image Analysis Software - Imaris - Oxford Instruments (oxinst.com)) and Huygens 20.04 (Huygens Software | Scientific Volume Imaging (svi.nl)) and figure compiled with Microsoft PowerPoint (Microsoft PowerPoint Slide Presentation Software | Microsoft 365). One mouse representative of 3 independent mice/experiments.

Supplementary Fig S11: *Positive identification of MAIT cells within WT BALB/c and CAST mice spleen with MR1-5-OP-RU tetramer in situ.*

Spleen sections from BALB/c WT (**a** to **c**) and CAST WT, CAST.MR1$^{-/-}$ (**d** to **e**) mice stained with MR1 tetramers loaded with 5-OP-RU or Ac-6-FP and anti-B220 and CD3 antibodies. **a** and **d**, Representative images showing MR1 tetramer (green), B220 (blue) and CD3 (red) staining within splenic Red Pulp (RP), B cell zone (BCZ) and T cell zone (TCZ) of the splenic white pulp, scale bar 100 μm with corresponding zoomed regions of interest as shown, scale bar 25 μm. **b** and **e** Count of MR1-5-OP-RU$^+$CD3$^+$ MAIT cells per 0.72 mm^2 image and **c** and **f** proportion of MAIT cells within each region of the total number of NKT cells per image, mean ± SEM. Microscopy images acquired with Zen 2012 (ZEISS ZEN Microscope Software for Microscope Components) and processed with FIJI/ImageJ (Fiji (imagej.net)) and Imaris 9.3 (Microscopy Image Analysis Software - Imaris - Oxford Instruments (oxinst.com)) and figure compiled with Microsoft PowerPoint (Microsoft PowerPoint Slide Presentation Software | Microsoft 365). Data n = 10 mice, 5 mice per experiment of 2 independent experiments, Wilcoxon test for data from BALB/c mice and Kruskal-Wallis with Dunn's post-hoc test for data from CAST mice, ** = $p < 0.01$.

Supplementary Fig S12: *CD1d and MR1 tetramer co-stain for NKT cells MAIT cells within WT C57BL/6 and BALB/c mice spleen in situ.*

Sections of spleens from C57BL/6 (**a**) and BALB/c (**b**) mice stained with CD1d-α-GalCer and MR1-5-OP-RU tetramers and anti-B220 and CD3 antibodies. **a** and **b**, Representative images showing CD1d tetramer (gray), MR1 tetramer (green), B220 (blue) and CD3 (red) staining within splenic red pulp (RP), B cell zone (BCZ) and T cell zone (TCZ) of the splenic white pulp, scale bar 100 μm with corresponding zoomed regions of interest as shown, scale bar 25μm. Microscopy images acquired with Zen 2012 (ZEISS ZEN Microscope Software for Microscope Components) and processed with FIJI/ImageJ (Fiji (imagej.net)) and

Imaris 9.3 (Microscopy Image Analysis Software - Imaris - Oxford Instruments (oxinst.com)) and figure compiled with Microsoft PowerPoint (Microsoft PowerPoint Slide Presentation Software | Microsoft 365). Images representative of n = 4 mice of 2 independent experiments. Tet = Tetramer.

Supplementary Fig S13: *No MAIT cells identified within C57BL/6, BALB/c and CAST mice thymus with MR1-5-OP-RU tetramer.*

Thymus sections from C57BL/6 WT and C57BL/6.MR1$^{-/-}$ (**a**), BALB/c WT (**b**) and CAST WT and CAST.MR1$^{-/-}$ (**C**) mice stained with MR1 tetramers loaded with 5-OP-RU or Ac-6-FP and anti-CD3 antibody and DAPI or anti-K5, CD205 and CD3 antibodies. Representative images showing (**a**) MR1 tetramer (green), CD3 (red) and DAPI (blue) staining, (**b**) MR1 tetramer (green), CD3 (red) and K5 (blue), and (**c**) MR1 tetramer (green), CD3 (red), K5 (blue) and CD205 (gray). Cortex (C) and Medulla (M), green arrows MR1-tetramer^{+} and CD3^{+}, white arrows MR1-tetramer^{+} and CD3^{-}. Scale bar 100 μm with corresponding zoomed regions of interest as shown, scale bar 25 μm. Microscopy images acquired with Zen 2012 (ZEISS ZEN Microscope Software for Microscope Components) and processed with FIJI/ImageJ (Fiji (imagej.net)) and figure compiled with Microsoft PowerPoint (Microsoft PowerPoint Slide Presentation Software | Microsoft 365). Images representative of 2 independent experiments with 2 mice each.

Supplementary Fig S14: T*he expansion of MAIT cells following intranasal administration of 5-OP-RU.*

C57BL/6.CAST WT mice were given 5-OP-RU intranasally or left unstimulated. On day 5 post antigen administration the mediastinal lymph nodes (mLN) were harvested and snap frozen. 7μm sections were cut and stained with B220 and CD3 antibodies and either 5-OP-RU- or Ac-6-FP-loaded MR1 tetramer. Representative images of (**a**) unstimulated and (**b**) stimulated mLN showing MR1 tetramer (green), B220 (blue) and CD3 (red) staining within the splenic paracortex (P), follicle (F), intrafollicular space (IF) and medulla (M), scale bar 100μm, with corresponding zoomed regions of interest, scale bar 25μm. **c** Total count of MAIT cells per 0.63 mm^2 image and **d** proportion of MAIT cells within each region at each time point. Microscopy images acquired with Zen 2012 (ZEISS ZEN Microscope Software for Microscope Components) and processed with FIJI/ImageJ (Fiji (imagej.net)) and Imaris 9.3 (Microscopy Image Analysis Software - Imaris - Oxford Instruments (oxinst.com)) and figure compiled with Microsoft PowerPoint (Microsoft PowerPoint Slide Presentation Software | Microsoft 365). Mean ± SEM. Data n = 3 mice of one experiment, two-way ANOVA with Sidak post-hoc test, ** = $p < 0.01$.
